# Ganglioside GM3 prevents high fat diet-induced hepatosteatosis via attenuated insulin signaling pathway

**DOI:** 10.1371/journal.pone.0281414

**Published:** 2023-02-24

**Authors:** Orie Tajima, Yuki Fujita, Yuhsuke Ohmi, Koichi Furukawa, Keiko Furukawa

**Affiliations:** 1 Department of Biomedical Sciences, Chubu University College of Life and Health Sciences, Kasugai, Japan; 2 Department of Clinical Laboratory, Aichi Medical University Hospital, Nagakute, Japan; 3 Department of Clinical Engineering, Chubu University College of Life and Health Sciences, Kasugai, Japan; Tohoku University, JAPAN

## Abstract

Gangliosides, sialic acid-containing glycosphingolipids, are widely involved in regulations of signal transductions to control cellular functions. It has been suggested that GM3, the simplest structure among gangliosides, is involved in insulin resistance, whereas it remains unclear whether insulin signaling diminished by GM3 actually aggravates the pathological conditions in metabolic disorders. Moreover, the functional roles of gangliosides in the regulation of insulin signaling have not yet been fully elucidated in liver or hepatocytes despite that it is one of the major insulin-sensitive organs. To understand physiological roles of GM3 in metabolic homeostasis in liver, we conducted a high fat diet (HFD) loading experiment using double knockout (DKO) mice of GM2/GD2 synthase and GD3 synthase, which lack all gangliosides except GM3, as well as wild-type (WT) mice. DKO mice were strikingly resistant to HFD-induced hepatosteatosis, and hepatic lipogenesis-related molecules including insulin signaling components were down-regulated in HFD-fed DKO. Furthermore, we established primary hepatocyte cultures from DKO and WT mice, and examined their responses to insulin *in vitro*. Following insulin stimulation, DKO hepatocytes expressing GM3 showed attenuated expression and/or activations in the downstream components compared with WT hepatocytes expressing GM2. While insulin stimulation induced lipogenic proteins in hepatocytes from both genotypes, their expression levels were lower in DKO than in WT hepatocytes after insulin treatment. All our findings suggest that the modified gangliosides, i.e., a shift to GM3 from GM2, might exert a suppressive effect on lipogenesis by attenuating insulin signaling at least in mouse hepatocytes, which might result in protection of HFD-induced hepatosteatosis.

## Introduction

Glycosphingolipids (GSLs) are constituents of eukaryotic cell membranes and considered to be involved in a number of cellular processes, regulating cell differentiation and activation [1, 2]. Since acidic GSLs are enriched in central nervous system in vertebrates, GSLs have been considered to play important roles in the development and function of nervous systems [3]. However, all cells in our body express various GSLs, and their expression patterns are strictly regulated in terms of quantity and quality. Therefore, they should widely exert regulatory functions of cells and tissues by repeating synthesis, membrane expression and degradation (or remodeling).

Although the synthesis of various GSLs depends on the expression of a distinctive set of glycosyltransferases localized mainly in Golgi apparatus [4], the biological significance of this extensive variability is still unclear. Genetic manipulation of glycosyltransferases responsible for the synthesis of GSLs is an extremely useful approach to reveal diverse roles of GSLs *in vivo*. We have generated various mutant mouse lines lacking a particular lineage of GSLs. In particular, mutant mice of various glycosyltransferases responsible for the synthesis of gangliosides, sialic acid-containing GSLs, have been systematically generated, and their pivotal roles in the maintenance and repair of nervous tissues have been demonstrated [5–8]. Double knockout (DKO) mice of GM2/GD2 synthase (GM2S) [5] and GD3 synthase (GD3S) [6] lacked all ganglio-series gangliosides including asialo-series, except GM3. They exhibited more serious phenotypes than each single KO, i.e., earlier-onset severe neurodegeneration and intense neurological dysfunctions [7, 9–13].

On the other hand, findings that gangliosides, especially GM3, contribute to the regulation of metabolic systems such as insulin signaling have been accumulating [14, 15]. It was reported that increased synthesis of cellular GM3 might be involved in tumor necrosis factor-α (TNF-α)-induced insulin resistance in cultured adipocytes [16]. In contrast, suppressing GM3 expression by genetic deletion of GM3 synthase (GM3S) or pharmacological inhibition of glycosyltransferases resulted in enhanced responses to insulin in some tissues of various rodent models [17–20]. While these findings suggest that GM3 may function as an inhibitor of insulin signaling, it is controversial whether insulin signaling diminished by GM3 actually aggravates the pathological conditions in metabolic disorders. Furthermore, the functional significance of GM3 in the regulation of insulin signaling in liver or hepatocytes has not yet been fully elucidated, while it is one of the major insulin-sensitive and lipogenic organs in the body.

DKO mice of GM2S and GD3S are a very beneficial model to directly verify the physiological roles of GM3 in the regulation of metabolic homeostasis, because they express GM3 alone among gangliosides through a whole-body. In this study, we revealed that DKO mice did not develop either obesity or hyperglycemia with aging. To further understand regulatory roles of GM3 in the metabolic homeostasis, we conducted a long-term high fat diet (HFD) loading experiment and explored alternations of metabolic status in DKO mice, especially focusing on liver. Since DKO mice were strikingly resistant to HFD-induced hepatosteatosis, the expression levels of molecules involved in lipid metabolism were rigorously analyzed. Furthermore, responses of cultured hepatocytes to insulin were examined by establishing primary hepatocyte cultures from DKO mice as well as wild-type (WT) mice, showing important roles of GM3 in the attenuation of insulin signaling and suppression of hepatic lipogenesis.

## Materials and methods

### Mice

DKO mutant mice of GM2S and GD3S were generated as described [7], and bred back to a pure C57BL/6 genetic background. Mice were kept under standard laboratory conditions of 12-h light followed by 12-h darkness. For the HFD loading experiments, male mice at 4 weeks of age were fed *ad libitum* either control diet (CD) (D12450B, Research Diets, New Brunswick, NJ, USA) containing 10 kcal% fat or HFD (D12492, Research Diets) containing 60 kcal% fat for 15 weeks. Body weight and food consumption were measured every week. After loading experiment finished, blood and tissue samples were collected. For tissue collections, unless specified, mouse was anesthetized with isoflurane and sacrificed by blood drawing from heart. The collected samples were immediately frozen in liquid nitrogen and stored at -80°C until each analysis. All experiments were performed following the guidelines of the Chubu University Committee on Animal Research and the Experimental Animal Care. When these guidelines were devised, the “Guide for the Care and Use of Laboratory Animals, 8th edition” [21] and “The ARRIVE guidelines 2.0” [22] were followed as well as the guideline from the Ministry of Education, Culture, Sports and Technology of Japan (MEXT).

### Glucose tolerance test (GTT) and insulin tolerance test (ITT)

For GTT, male mice at 15–20 weeks and 35–40 weeks of age were administered glucose (2g/Kg) by intraperitoneal (IP) injection following a 16-h fasting. For ITT, mice were administered insulin (0.75U/Kg) by IP injection following a 4-h fasting. Blood glucose were measured at 0, 15, 30, 60, 120, and 180 min after the individual injection using OneTouch^TM^ UltraVue™ blood glucose meter (Johnson & Johnson, New Brunswick, NJ, USA).

### Blood parameters

Following a 16-h fasting, glucose levels in blood collected from the tail vein were measured using the blood glucose meter. Serum levels of triglycerides (TG), non-esterified fatty acids (NEFA), and cholesterol were quantified using a Triglyceride E-test^TM^ Wako kit (Wako, Osaka, Japan), a NEFA C-test^TM^ Wako kit (Wako), and a Cholesterol E-test^TM^ Wako kit (Wako), respectively.

### Histology

Mouse was anesthetized with isoflurane and perfused through the left cardiac ventricle with PBS, followed by 4% paraformaldehyde in 0.1 M sodium phosphate buffer (pH 7.4). Liver and epididymal fat were dissected and fixed in 4% paraformaldehyde solution overnight. The samples were embedded in paraffin, and 4-μm paraffin sections were stained with hematoxylin and eosin. For oil red staining, liver samples were embedded in optimal cutting temperature compound (OCT), and sectioned on a cryostat at a 10-μm thickness.

### Extraction of GSLs and thin layer chromatography (TLC)

Liver lipids were extracted with chloroform/methanol (2:1, v/v), and sequentially re-extracted with chloroform/methanol (1:1, v/v) and chloroform/methanol (1:2, v/v). Supernatants were pooled, dried, and subjected to mild saponification in 0.1 M NaOH in chloroform/methanol (1:1) at 37°C for 2 h and then evaporated to dryness. Samples were reconstituted in chloroform /water (1:1, v/v) and applied to a C18 Sep-Pak cartridge (Waters, Milford, MA, USA) equilibrated in the same solvent system. After washing with chloroform/water (1:1, v/v), GSLs were eluted by chloroform/methanol (1:1, v/v) and then chloroform/methanol (1:2, v/v) sequentially. Extracted GSLs were fractionated to neutral and acidic fractions using the DEAE-sephadex^TM^ column. Acidic lipid fraction was dissolved in chloroform/methanol (1:1, v/v), and a fraction equivalent to 50 mg of wet tissue was applied to a silica-gel-coated plate. TLC was performed with chloroform/methanol/ 0.2% CaCl_2_ (55: 45: 10) and bands were detected by resorcinol spray.

### Quantification of lipids in livers

Liver lipids were extracted with chloroform/methanol (2:1, v/v), and sequentially re-extracted with chloroform/methanol (1:1, v/v). Hepatic contents of TG, NAFA, and cholesterol were quantified using Triglyceride E-test^TM^ Wako kit (Wako), NEFA C-test^TM^ Wako kit (Wako), and Cholesterol E-test^TM^ Wako kit (Wako), respectively.

### Real-time reverse transcription-polymerase chain reaction (RT-PCR)

Total RNA was extracted from livers of mice using the RNeasy Lipid Tissue Mini^TM^ kit (Qiagen, Hilden, Germany) in accordance with the manufacturer’s protocol. To synthesize cDNA, reverse transcription was performed using 2 μg of total RNA, and the High Capacity cDNA Reverse Transcription^TM^ kit (Applied Biosystems, Foster City, CA, USA) in a total volume of 20 μL. Real-time RT-PCR reactions were performed using Applied Biosystems 7300^TM^ with SYBR Green Master Mix^TM^ (Applied Biosystems). Relative quantification of gene expression was analyzed by the 2^-ΔCt^ method, and values of expression levels of mRNAs were normalized with internal control gene glyceraldehyde-3-phosphate dehydrogenase (GAPDH) examined in parallel. Primer sequences used for real-time RT-PCR are listed in S1 Table.

### Western immunoblotting

Liver and isolated hepatocytes were homogenized in RIPA buffer containing a protease inhibitor cocktail (Thermo Scientific, Waltham, MA, USA) and incubated on ice for 10 min. After centrifuging for 15 min at a maximum speed, the protein concentration of the supernatant was determined using the Pierce^TM^ BCA Protein Assay Kit (Thermo Fisher Scientific). Twenty micrograms of liver protein or 5 μg of hepatocyte protein were used for immunoblotting with primary antibodies for fatty acid translocase (FAT/CD36) (AF2519, R&D Systems, Minneapolis, MN, USA), fatty acid binding protein 1 (FABP1) (ab7366, Abcam), fatty acid synthase (FASN) (3180, Cell Signaling Technology, Beverly, MA, USA), acetyl-CoA carboxylase (ACC) (3676, Cell Signaling Technology), insulin receptor (IR) (3025, Cell Signaling Technology), PY99 (p-Tyr, 7020, Santa Cruz Biotechnology, Dallas, TX, USA), insulin receptor substrate-1 (IRS-1) (611394, BD Bioscience, Franklin Lakes, NJ, USA), phosphatidylinositol-3-kinase (PI3-K) (610045, BD Bioscience), sterol regulatory element-binding protein-1c (SREBP-1c) (NB600-582, NB100-2215, Novus Biologicals, Centennial, CO, USA), protein kinase B (Akt) (9272, Cell Signaling Technology), phospho-Akt (Ser473, 9271, Cell Signaling Technology), mammalian target of rapamycin (mTOR) (2983, Cell Signaling Technology), phospho-mTOR (Ser2448, 2971, Cell Signaling Technology), AMP-activated protein kinase α (AMPKα) (2603, Cell Signaling Technology), phospho-AMPKα (Thr172, 2535, Cell Signaling Technology), or β-Actin (A5441, Sigma-Aldrich, St. Louis, MO, USA), at dilutions suggested by the manufacturers. Densitometric analysis was performed by ImageQuant^TM^ LAS 4000 (GE Healthcare Bioscience, Piscataway, NJ, USA), and quantification values were normalized against β-Actin.

### Primary hepatocyte isolation and *in vitro* insulin stimulation

Primary hepatocytes were isolated from mice at 15 weeks of age. In brief, mouse was anesthetized with isoflurane and the liver was perfused *in situ* through the hepatic portal vein with 0.5 mM EGTA/PBS at a rate of 5 mL/min for 5 min followed by perfusion with collagenase-containing solution [Dulbecco’s modified Eagle’s medium (DMEM) with 0.05% Collagenase Type Ⅳ (Worthington Bioscience, Lakewood, NJ, USA)] until complete digestion. The liver was carefully removed, transferred into Petri dishes with collagenase-containing solution, and mechanically dissociated. Ten mL of ice-cold DMEM supplemented with 10% fetal bovine serum (FCS) was added to cells, and the liver fractions were filtered through a 70-μm nylon cell strainer (BD Bioscience) and centrifuged at 100 × g for 3 min at 4°C. The pellets were collected as hepatocytes. After washing 3 times with 10% FCS/DMEM, freshly isolated hepatocytes were suspended in 10% FCS/DMEM and immediately plated on collagen-coated dishes. After overnight culture in DMEM with or without 10% FCS, the hepatocytes were treated with 10 nM insulin and sampled at 5, 60, and 360 min later.

### Statistical analysis

All results are presented as means ± standard deviation (S.D.). Differences between WT and DKO mice were analyzed by the two-tailed Student’s t-test. A *P*-value of less than 0.05 was considered to be significant.

## Results

### DKO mice were protected from age-induced obesity with normal blood glucose level

**Fig 1:**
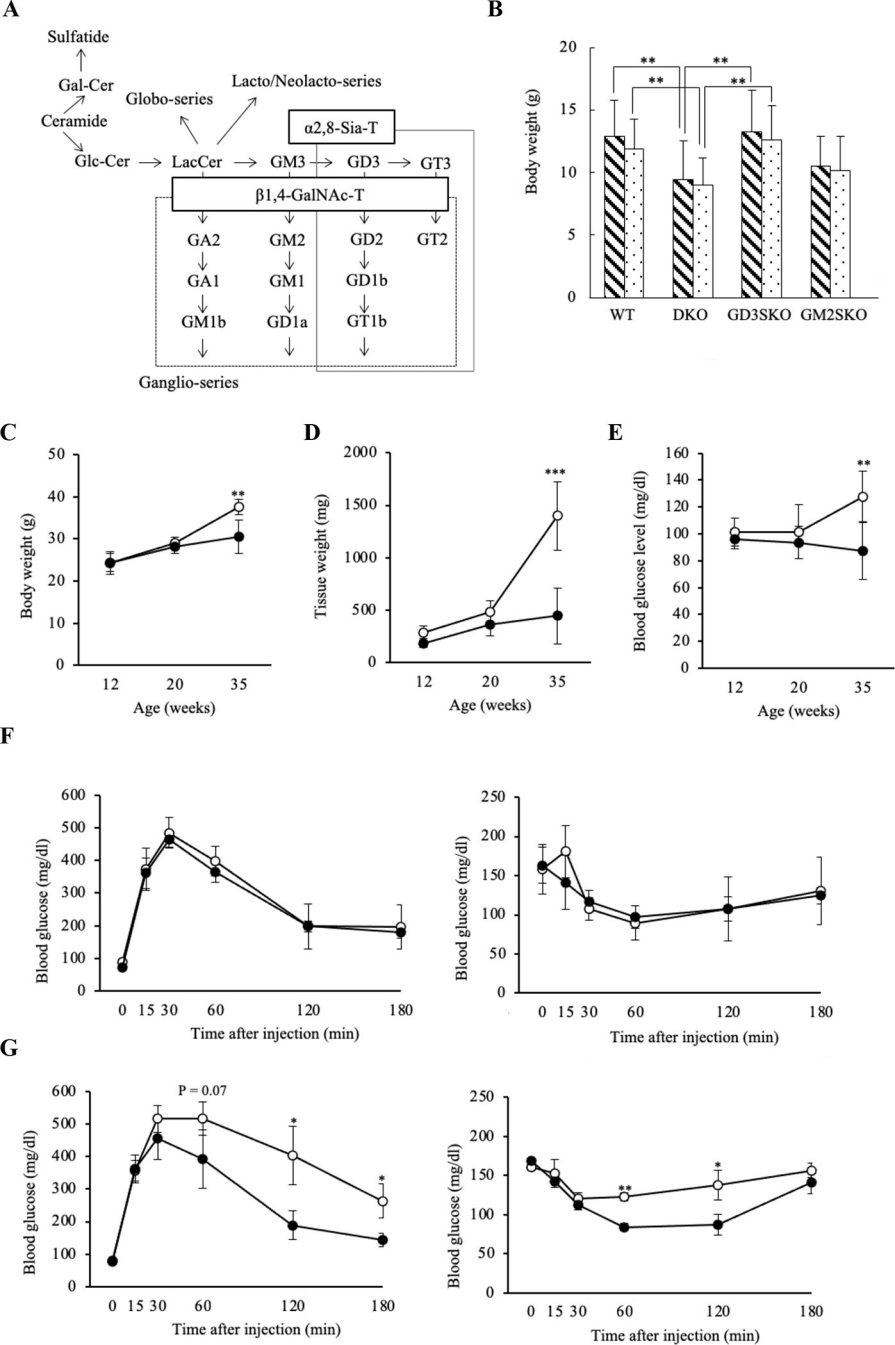
DKO mice expressing only GM3 did not show obesity and hyperglycemia. (**A**) A synthetic pathway of gangliosides. Ganglioside species that are absent in KO mice for GM2S (β1,4-GalNAc-T) and GD3S (α2,8-Sia-T) genes are enclosed by dotted line and solid line, respectively. Consequently, all structures boxed in two squares were depleted in DKO mice. (**B**) Body weights at 4 weeks of age in ganglioside-deficient mouse lines (more than 20 mice each). Oblique line and dot bars show male and female mice, respectively. Data are expressed as means ± S.D. **P<0.01 as compared with WT or GD3S-KO and DKO mice. (**C**) Body weights, (**D**) adipose tissue weights isolated from epididymal fat, and (**E**) blood glucose levels at 12 (3 mice each), 20 (5 mice each), and 35 weeks of age (6 mice each). Open and closed circles show WT and DKO mice, respectively. Data are expressed as means ± S.D. **P<0.01 and ***P<0.001 as compared between WT and DKO mice. (**F**) Blood glucose levels during GTT (left) and ITT (right) at 20 weeks of age (3 mice each). Open and closed circles show results of WT and DKO mice, respectively. Data are expressed as means ± S.D. (**G**) Blood glucose levels during GTT (left) and ITT (right) at 35–40 weeks of age (3 mice each). Open and closed circles show results of WT and DKO mice, respectively. Data are expressed as means ± S.D. *P<0.05 and **P<0.01 as compared between WT and DKO mice.

DKO mice were generated by mating GM2S-KO and GD3S-KO mice and they were unable to synthesize all ganglio-series gangliosides except GM3 (Fig 1A). The body weights of DKO mice were lower than those of WT mice or other single gene mutants of ganglioside synthases at 4 weeks of age (Fig 1B). In the follow-up of progressively aging mice, DKO mice showed reduced whole-body (Fig 1C) and epididymal fat (Fig 1D) weights at 35 weeks of age compared with age-matched WT mice. Indirect calorimetry studies revealed that metabolic parameters were similar between DKO and WT mice in both younger (20 weeks) and older (35–40 weeks) groups (S1A and S1B Fig). There were no significant differences in energy intake during 24 h between WT and DKO mice (S1C Fig). These results indicate that DKO mice systemically expressing only GM3 did not develop age-induced obesity without increased energy consumption or decreased energy intake.

Furthermore, fasting blood glucose levels were significantly lower in aged DKO than in WT mice (Fig 1E). Although previous reports suggested that GM3 might act as an inducer of insulin resistance [14, 15], DKO mice systemically expressing only GM3 showed normal control of blood glucose level as analyzed by GTT and ITT at 20 weeks of age (Fig 1F). These results reveal that only GM3 expression does not necessarily result in systemic insulin resistance. More surprisingly, older DKO mice aged at 35–40 weeks old maintained glucose tolerance though age-matched WT showed glucose intolerance (Fig 1G). Consequently, GM3 might be important for maintenance of systemic glucose homeostasis.

### DKO mice showed attenuated HFD-induced obesity and obesity-related metabolic disorders

**Fig 2:**
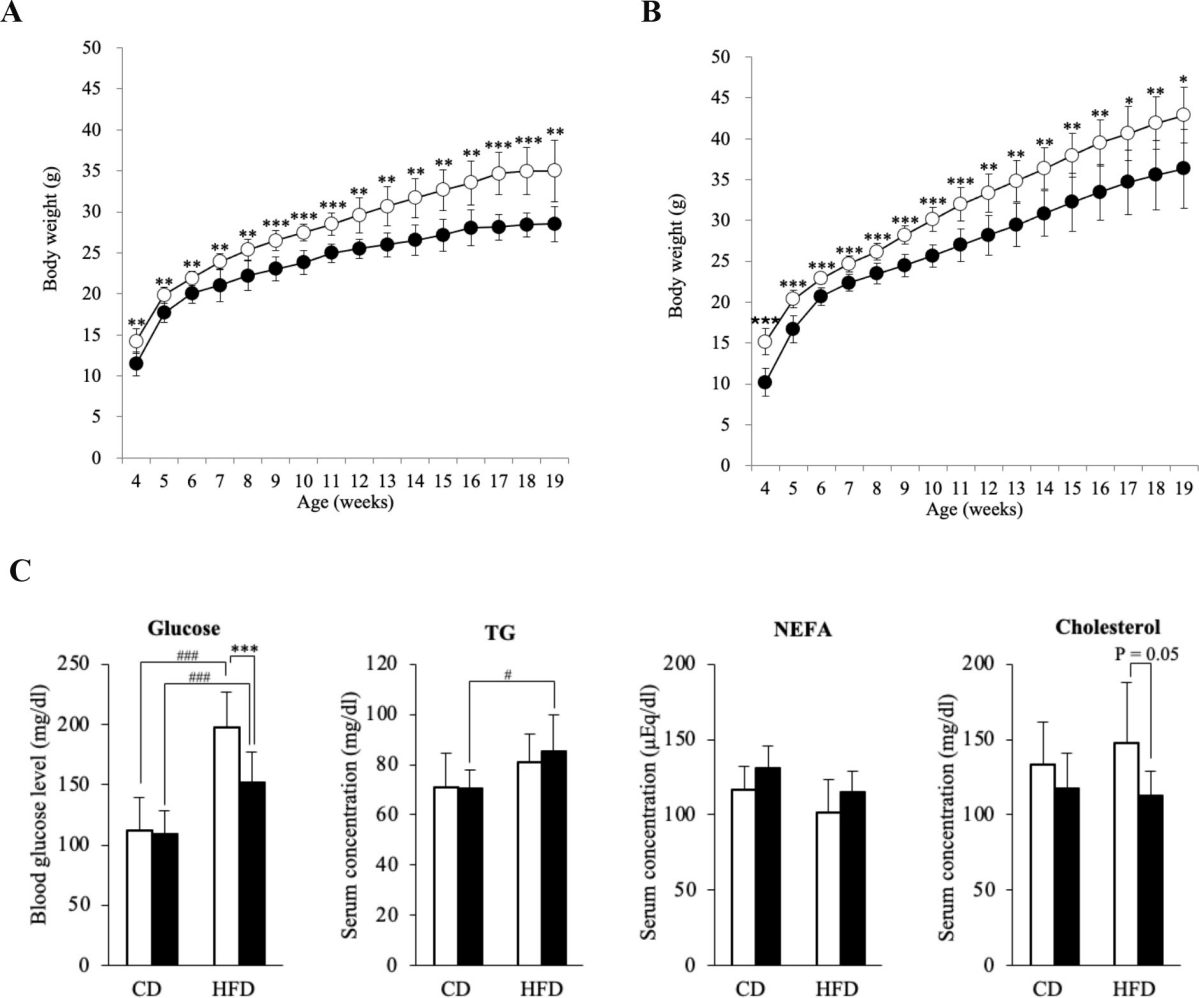
DKO mice showed milder phenotypes in HFD-induced body weight gain and hyperglycemia. (**A**) Body weights of WT and DKO mice in CD-fed states (WT, *n* = 7; DKO, *n* = 6), and (**B**) HDF-fed states (7 mice each). Body weights recorded every week from 4 to 19 weeks old. Open and closed bars indicate WT and DKO mice, respectively. Data are expressed as means ± S.D. *P<0.05, **P<0.01, and ***P<0.001 as compared between WT and DKO mice. (**C**) Blood glucose and serum levels of lipids in CD-fed or HFD-fed mice. Open and closed bars show WT and DKO mice, respectively. Data are expressed as means ± S.D. ***P<0.001 as compared between WT and DKO mice. ^#^P<0.05 and ^###^P<0.001 as compared between CD and HFD groups.

To further understand the alternations of metabolic status in DKO mice, male mice were placed on a CD or HFD for 15 weeks from 4 weeks after birth. There were no differences in daily energy intake between WT and DKO mice in the HFD group, though DKO mice showed slightly more intake than WT mice in the CD group (S2A Fig). In the CD group, DKO mice showed significantly lower body weights than WT mice during the whole experimental period (Fig 2A). HFD-fed DKO mice as well as HFD-fed WT showed marked weight gain, although DKO mice showed about a 15% lower body weights than WT mice after the end of the 15-week HFD loading experiment (Fig 2B). Weights of epididymal fat were significantly lower in DKO than WT mice regardless of diet groups (S2B Fig). Increased adiposity with HFD feeding were shown in both genotypes (S2C Fig), whereas DKO adipocytes were significantly smaller than WT adipocytes (S2D Fig). Moreover, HFD loading increased fasting blood glucose levels in mice with both genotypes, whereas HFD-fed DKO mice showed significantly lower blood glucose than HFD-fed WT mice (Fig 2C). Serum cholesterol levels were lower in HFD-fed DKO mice than HFD-fed WT mice (Fig 2C). Thus, these findings strongly indicate that systemic expression of GM3 attenuated HFD-induced obesity and obesity-related phenotypes including hyperglycemia and hypercholesterolemia.

### DKO mice were resistant to HFD-induced hepatosteatosis with GM3 expression in liver

**Fig 3:**
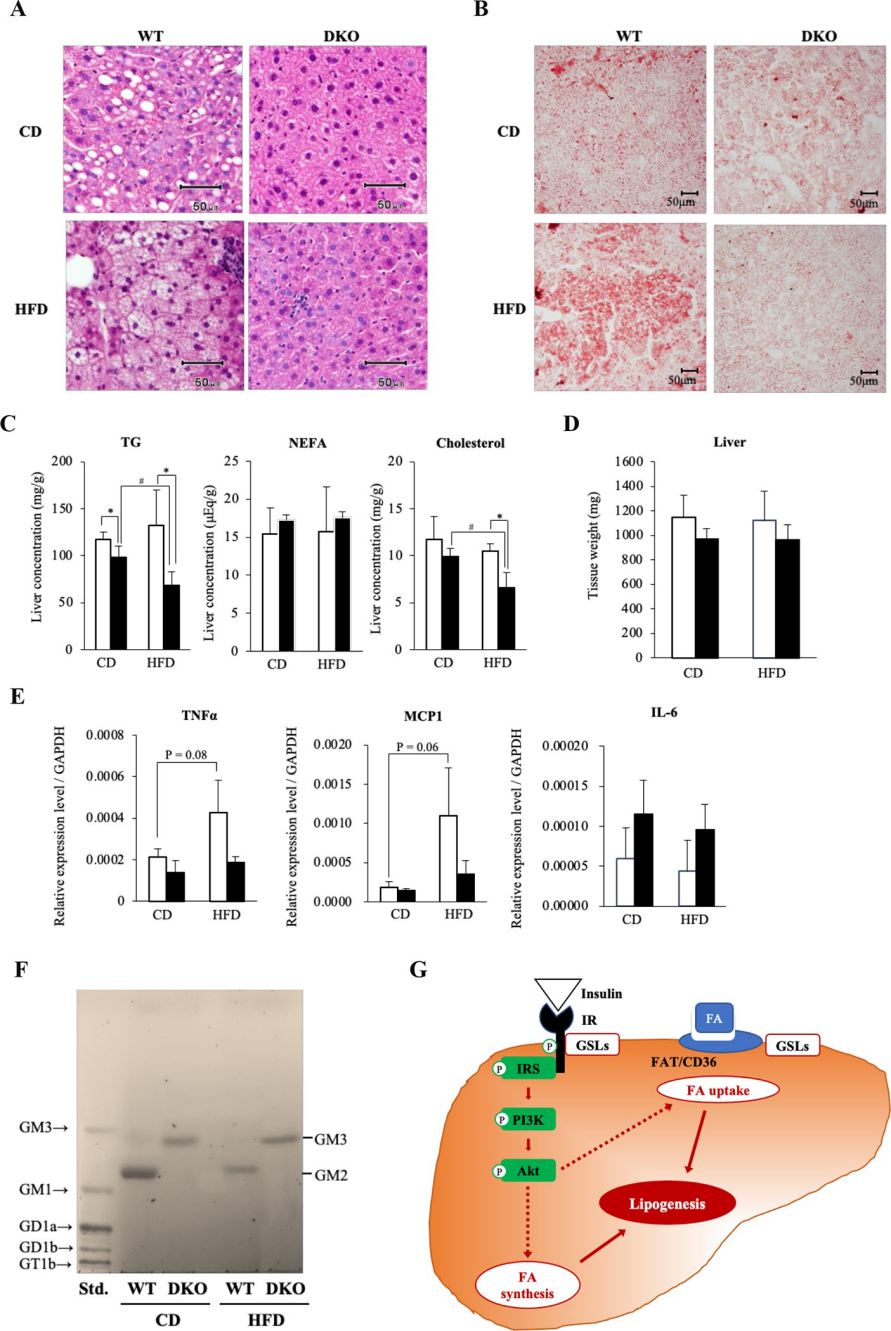
DKO mice were resistant to HFD-induced hepatosteatosis with modified gangliosides, a shift to GM3 from GM2, in liver. (**A**) Hematoxylin and eosin staining, and (**B**) Oil red staining of livers of CD-fed (upper) or HFD-fed (lower) mice. Scale bars represent 50 μm. (**C**) Hepatic lipid levels, and (**D**) Tissue weights of livers of CD-fed (4 mice each) or HFD-fed (WT, *n* = 3; DKO, *n* = 4) mice. Open and closed bars show WT and DKO mice, respectively. Data are expressed as means ± S.D. *P<0.05 as compared between WT and DKO mice. ^#^P<0.05 as compared between CD and HFD groups. (**E**) mRNA levels of cytokines in the liver normalized with GAPDH. Open and closed bars indicate WT and DKO mice, respectively. The numbers of mice examined were: WT, *n* = 3; DKO, *n* = 3 in the individual diet groups. Data are expressed as means ± S.D. (**F**) TLC of acidic glycolipids of livers. Detected bands are estimated as GM3 (upper) and GM2 (lower). Gangliosides derived from 50-mg liver tissue samples were applied to individual lanes. Bovine brain ganglioside mixture was used as a standard (std). (**G**) A schema to show possible mechanisms of altered hepatosteatosis due to ganglioside changes. Gangliosides might regulate the insulin signaling pathway to control lipogenesis including fatty acid synthesis and uptake. IR, insulin receptor; IRS, insulin receptor substrate; FA, fatty acid.

As for liver, histological analysis revealed that the presence of many lipid droplets was recognized in CD-fed WT mice (Fig 3A). HFD loading led to marked lipid storage in WT livers, whereas DKO livers scarcely showed lipid accumulation (Fig 3A and 3B). These histologic phenotypes were reflected in the result that the TG concentration was increased more in WT livers than DKO livers in both diet groups (Fig 3C). Liver TG and cholesterol levels were decreased by about 50 and 40%, respectively, in HFD-fed DKO mice compared with those in WT mice (Fig 3C), though there were no differences in liver weights between WT and DKO mice in both diet groups (Fig 3D). These findings indicate that DKO mice were resistant to HFD-induced hepatosteatosis. Since hepatosteatosis was frequently associated with inflammation, we further investigated gene expression levels of inflammatory cytokines in livers using real-time RT-PCR. TNF-α and monocyte chemotactic protein 1 (MCP1), but not interleukin-6 (IL-6), were markedly up-regulated in HFD-fed WT mice, while those cytokines did not increase in livers of HFD-fed DKO mice (Fig 3E). These findings were consistent with the histologic status showing minimal hepatic lipid accumulation in DKO mice.

To analyze changes in GSL composition, we performed TLC of extracted glycolipids from livers after the CD or HFD loading experiment. A major ganglioside was GM2 in CD-fed WT mouse livers, while CD-fed DKO mice expressed only GM3 as expected because of the deficiency of GM2S and GD3S (Figs 1A and 3F). Although expressed gangliosides did not change after HFD loading both in WT and DKO mice, the amounts of GM2 per tissue weight markedly decreased in the HFD group compared with the CD group in WT livers (Fig 3F).

The mechanisms of altered hepatosteatosis due to ganglioside changes became the focus of further experiments at this stage (Fig 3G). Namely, regulation of insulin signals with individual gangliosides were intensively investigated hereafter.

### Lipogenesis-related molecules were down-regulated in livers of HFD-fed DKO mice

**Fig 4:**
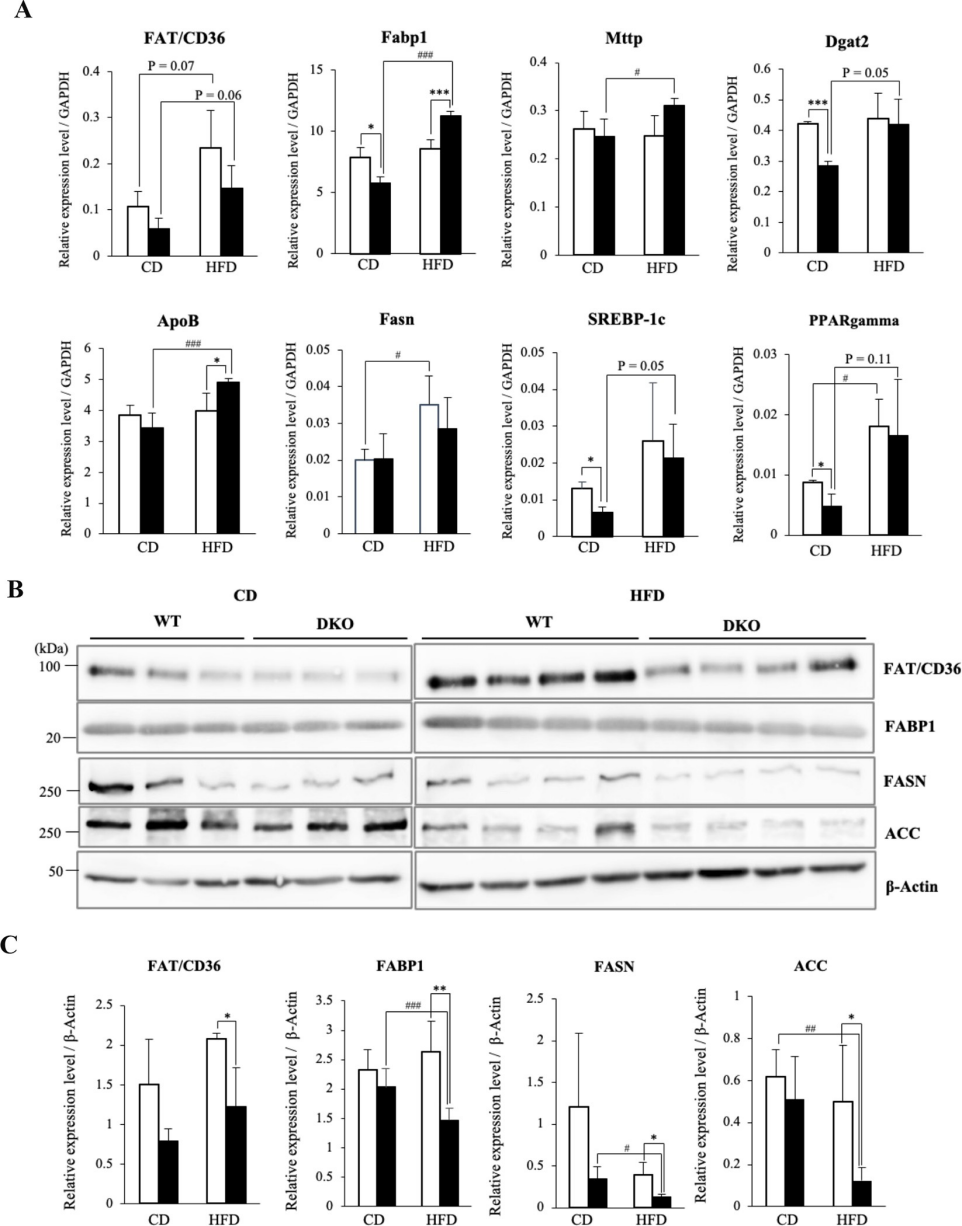
Expressions levels of hepatic lipogenesis-related molecules were lower in HFD-fed DKO mice than in WT mice. (**A**) mRNA levels of lipid metabolism-related genes in livers of CD-fed or HFD-fed mice were analyzed by real-time RT-PCR, and are presented after correction with the GAPDH gene. Open and closed bars indicate WT and DKO mice, respectively. The numbers of mice examined were WT, *n* = 3; DKO, *n* = 3 in individual diet groups. Data are expressed as means ± S.D. *P<0.05 and ***P<0.001 as compared between WT and DKO mice. ^#^P<0.05 and ^###^P<0.001 as compared between CD and HFD groups. (**B**) Protein levels of lipid metabolism-related molecules in livers of CD-fed (3 each) or HFD-fed (4 each) mice were analyzed by immunoblotting. (**C**) Relative expression levels are presented by measuring band intensities in (B) after correction with those of β-Actin. Open and closed bars indicate WT and DKO mice, respectively. Data are expressed as means ± S.D. *P<0.05 and **P<0.01 as compared between WT and DKO mice. ^#^P<0.05, ^##^P<0.01 and ^###^P<0.001 as compared between CD and HFD groups.

To gain insight into the molecular mechanisms of resistance to HFD-induced hepatosteatosis in DKO mice, we performed real-time RT-PCR to examine the expression levels of genes implicated in lipid metabolism in liver. Among 8 genes examined, major transcriptional regulators of lipid metabolism, SREBP-1c and peroxisome proliferator-activated receptor gamma (PPAR gamma), were significantly decreased in DKO mouse livers in the CD group (Fig 4A). The expression levels of Fabp1, involved in fatty acid uptake and intracellular transport, and diacylglycerol acyltransferase 2 (Dgat2), an enzyme catalyzing the final step of TG synthesis, were also lower in CD-fed DKO, suggesting that the livers of DKO mice showed reduced lipid metabolism in the steady state. In DKO livers, examined all genes, except Fasn, were up-regulated after HFD loading (Fig 4A). However, proteins involved in uptake and synthesis of fatty acids were down-regulated after HFD in DKO livers (Fig 4B and 4C), suggesting that up-regulations of these genes were compensated effects for lower proteins in HFD.

In HFD-fed mice, the expressions of FAT/CD36 or Fasn were up-regulated in both genotypes compared with CD-fed mice, while these levels were slightly lower in HFD-fed DKO livers than in WT (Fig 4A). As for proteins involved in uptake and synthesis of fatty acids, we further revealed that not only FAT/CD36 and FASN but also FABP1 and ACC were significantly lower in DKO livers than in WT (Fig 4B and 4C).

**Fig 5:**
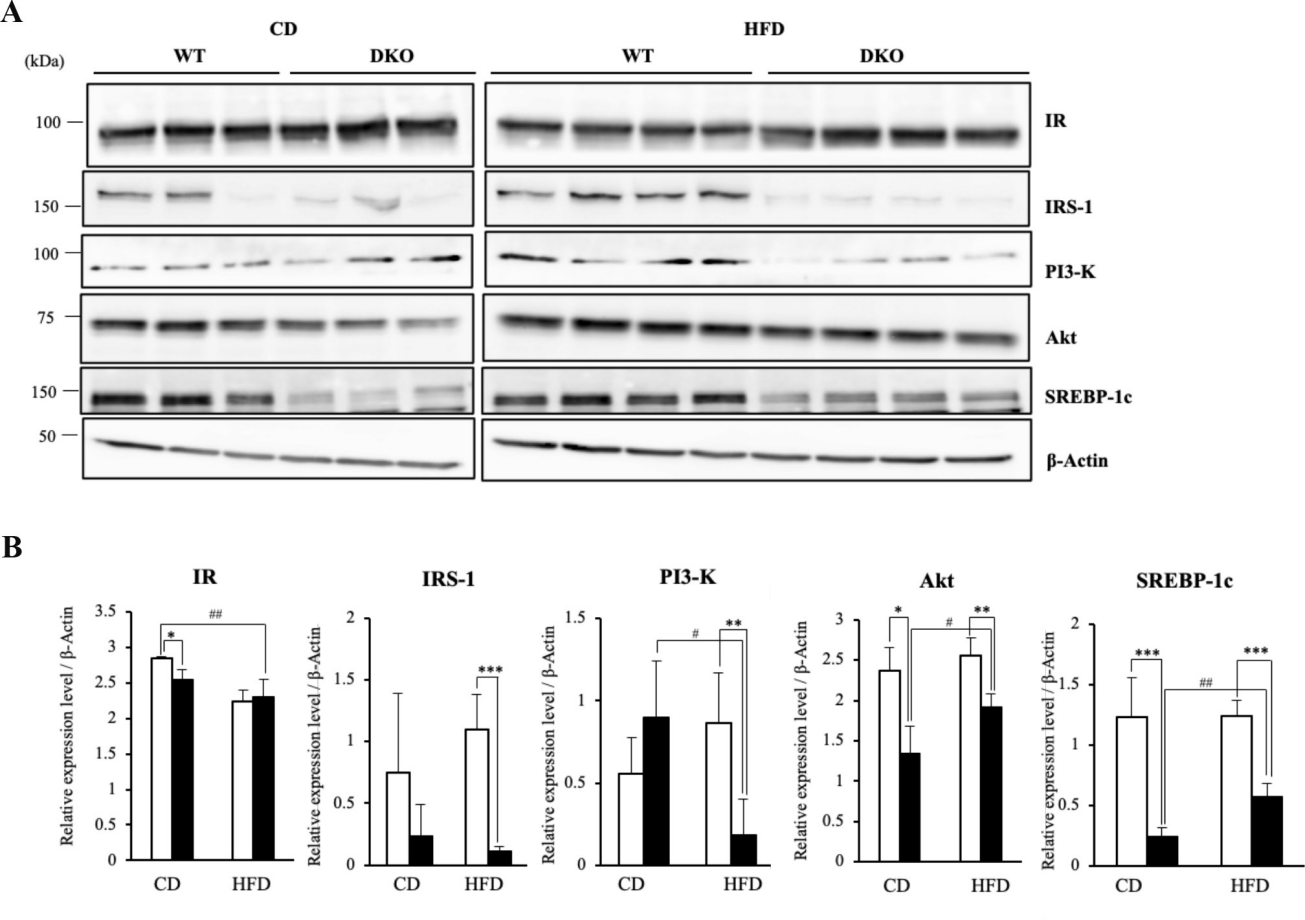
Expression levels of insulin signaling pathway-related proteins were reduced in livers of HFD-fed DKO mice compared with WT mice. (**A**) Protein levels of insulin signaling pathway-related molecules in livers of CD-fed (3 mice each) or HFD-fed (4 mice each) mice were analyzed by immunoblotting. (**B**) Relative expression levels are presented by measuring band intensities after correction with those of β-Actin. Open and closed bars indicate WT and DKO mice, respectively. Data are expressed as means ± S.D. *P<0.05, **P<0.01 and ***P<0.001 as compared between WT and DKO mice. ^#^P<0.05 and ^##^P<0.01 as compared between CD and HFD groups.

Because hepatic lipogenesis, including uptake and synthesis of fatty acids, is activated primarily by insulin [23], we further examined the expressions of proteins related to the insulin signaling pathway. While expression levels of IR were not different between WT and DKO mice in both feeding groups, the livers of DKO mice showed marked reductions of IRS-1, PI3-K, Akt and SREBP-1c compared with WT mice after HFD loading (Fig 5A and 5B), suggesting that the down-regulation of hepatic lipogenesis-related molecules was caused via the reduced components in the insulin signaling pathway in HFD-fed DKO mice.

### DKO hepatocytes expressing GM3 showed attenuated induction of insulin-mediated lipogenic molecules *in vitro*

To examine whether ganglioside modification affects hepatic insulin signaling, we established a primary culture of hepatocytes from DKO as well as WT mice and assessed responses to insulin stimulation *in vitro*. In serum starved condition, IR, Akt, and mTOR which constitute insulin signal cascade, were immediately phosphorylated after insulin stimulation in both hepatocytes (S3A Fig). Expression levels of phosphorylated IR, as well as phosphorylation levels evaluated by ratios of phosphorylated forms to total amounts, were slightly lower in DKO hepatocytes at 5 min after insulin stimulation, though those of Akt and mTOR were not different between WT and DKO (S3B and S3C Fig). While phosphorylated forms of AMPK, a cellular energy sensor, were markedly higher in DKO than WT hepatocytes before insulin stimulation, there were no significant differences of those levels between genotypes after insulin stimulation (S3A–S3C Fig).

**Fig 6:**
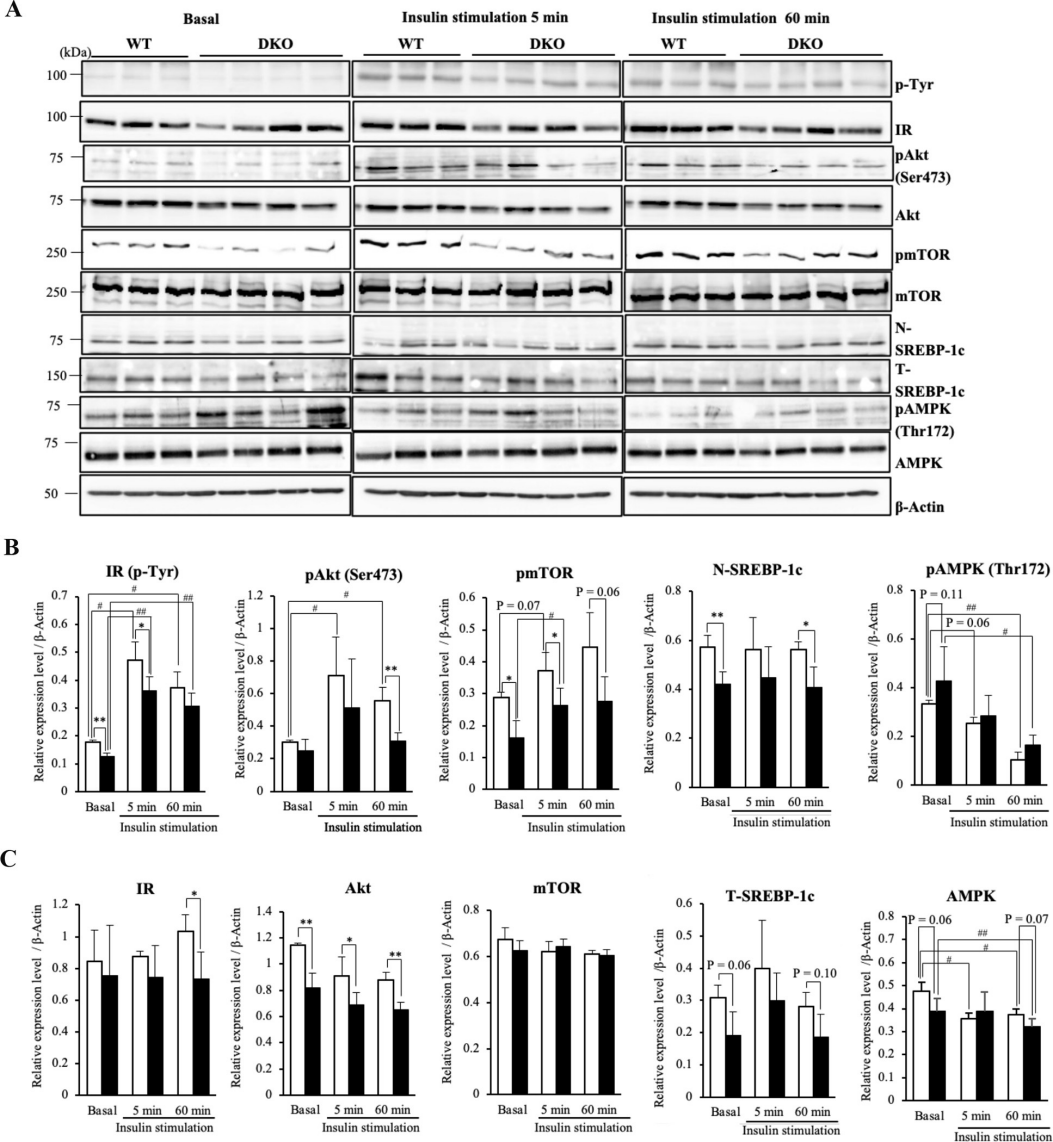
Insulin signaling was attenuated in primary culture DKO hepatocytes. (**A**) Primary culture hepatocytes were prepared, and stimulated by insulin as described in Materials and Methods. Activation levels of insulin signaling molecules in hepatocytes were analyzed by immunoblotting. p-Tyr showed results with Ab PY99 at IR site. (**B**) Relative expression levels of activated forms, and (**C**) total amounts of signaling molecules are presented by measuring band intensities after correction with those of β-Actin. The numbers of mice examined were WT, *n* = 3; DKO, *n* = 4. Data are expressed as means ± S.D. *P<0.05 and **P<0.01 as compared between WT and DKO hepatocytes. ^#^P<0.05 and ^##^P<0.01 as compared between basal and insulin stimulation.

To clarify the responses to insulin in cellular basal physiological condition, we further studied downstream activations of insulin signal cascade after overnight culture in serum plus medium. Phosphorylated forms of IR were immediately increased after insulin stimulation in both hepatocytes (Fig 6A). However, DKO hepatocytes already showed lower expressions of phosphorylated forms of IR at basal state (Fig 6B), and the reduced expressions also observed at 5 min after insulin stimulation (Fig 6B). Insulin stimulation significantly induced phosphorylation of Akt in WT hepatocytes but not in DKO hepatocytes (Fig 6A, 6B and S4 Fig), then expression levels of phosphorylated forms and phosphorylation levels of Akt were significantly lower in DKO than WT at 60 min after insulin stimulation (Fig 6B, S4 Fig). Although mTOR, located at downstream of IR and Akt in the insulin signaling pathway, was also phosphorylated by insulin stimulation after 5 min, phosphorylated forms and phosphorylation levels were significantly reduced in DKO hepatocytes compared with WT regardless of the insulin stimulation (Fig 6A, 6B and S4 Fig). As mTOR phosphorylation promotes lipid synthesis by activating a transcription factor SREBP-1c [24, 25], we analyzed the amounts of the precursor (T-SREBP-1c) and cleaved nuclear active forms (N-SREPB-1c). While significant activations by insulin could not be detected within 60 min in both hepatocytes (Fig 6A and S4 Fig), expression levels of N-SREBP-1c were significantly lower in DKO hepatocytes both with or without insulin (Fig 6B). Expression levels of total IR were not different between hepatocytes from WT and those from DKO mice at basal condition, nevertheless those were significantly lower in DKO than WT hepatocytes at 60 min after insulin stimulation (Fig 6C). Consistent with results of *in vivo* study, DKO hepatocytes showed lower expressions of total Akt and T-SREBP-1c with or without insulin stimulation (Fig 6C). These results indicate that DKO hepatocytes could respond to insulin but expression and/or activation of signaling molecules were reduced. Furthermore, insulin immediately reduced the phosphorylated forms and total amount of AMPK in WT hepatocytes, though the reductions of AMPK phosphorylation by insulin were milder in DKO hepatocytes (Fig 6A–6C). These results suggested that GM3 might attenuate insulin signaling in mouse hepatocytes cultured in cellular physiological condition with serum.

**Fig 7:**
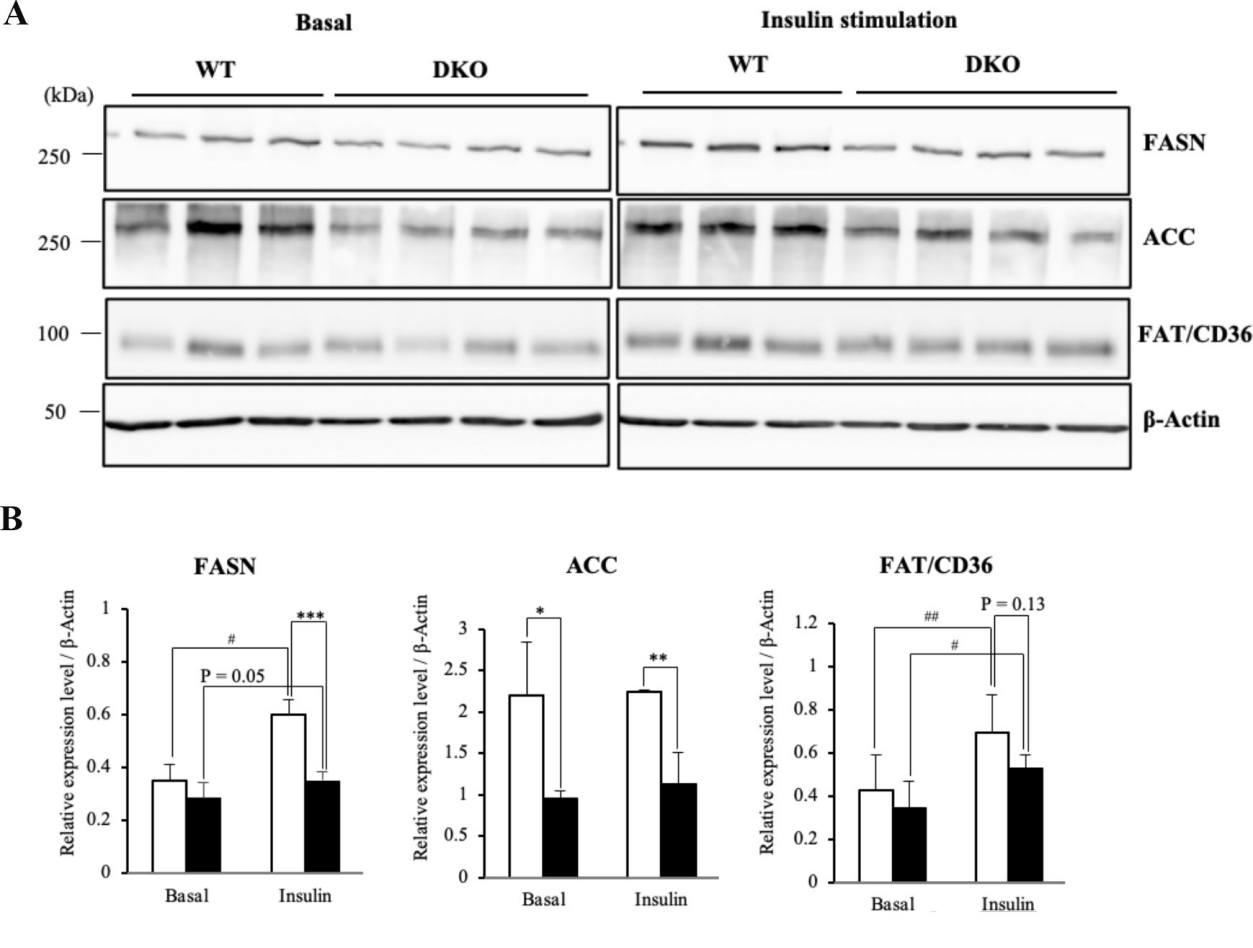
Induced levels of lipogenic proteins after insulin stimulation were lower in DKO hepatocytes. (**A)** Expression levels of FASN, ACC, and FAT/CD36 in hepatocytes at 360 min after insulin stimulation were analyzed by immunoblotting. (**B**) Expression levels of lipogenic molecules are presented by measuring band intensities after correction with those of β-Actin. Open and closed bars indicate WT and DKO hepatocytes, respectively. The numbers of mice examined were WT, *n* = 3; DKO, *n* = 4. Data are expressed as means ± S.D. *P<0.05, **P<0.01 and ***P<0.001 as compared between WT and DKO hepatocytes. ^#^P<0.05 and ^##^P<0.01 as compared between basal and insulin stimulation.

**Fig 8:**
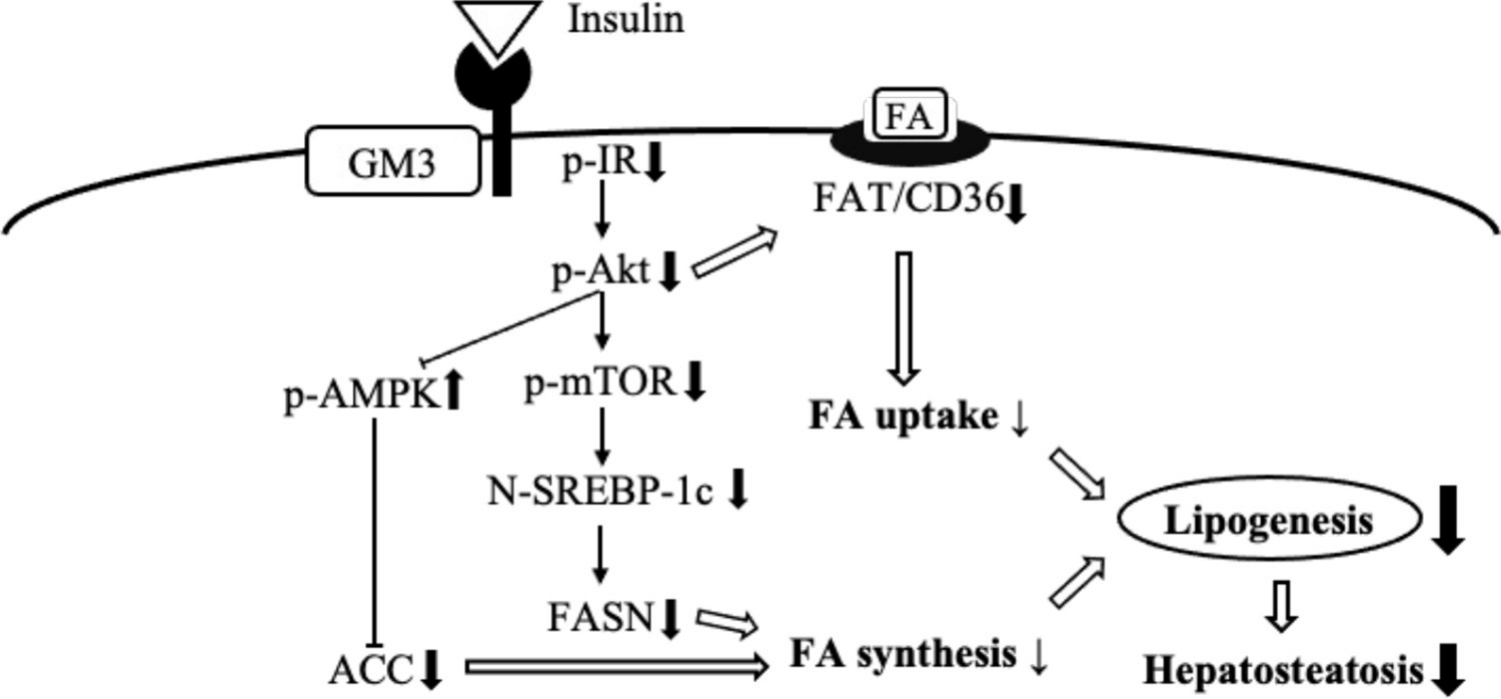
Regulatory roles of GM3 in hepatic insulin signaling, leading to the protection from hepatosteatosis. A schema to summarize the results of this study.

We also examined whether lipogenic proteins were induced by insulin in hepatocytes. FASN, known as one target of SREBP-1c [26], was up-regulated in hepatocytes from both genotypes after insulin treatment (Fig 7A and 7B), but the expression levels of FASN in DKO hepatocytes were lower at about 40% of that of WT after insulin treatment (Fig 7B). The expression levels of ACC, another target of SREBP-1c [26], were markedly reduced in DKO hepatocytes regardless of insulin stimulation (Fig 7B), whereas insulin stimulation did not enhance ACC in either genotype of hepatocytes (Fig 7A and 7B). While insulin stimulation induced FAT/CD36, which does not possess SREBP-1c binding element, in hepatocytes from both genotypes (Fig 7A and 7B), the expression level of FAT/CD36 was about 25% lower in DKO than WT hepatocytes after insulin treatment (Fig 7B). All these results suggest that the altered gangliosides in hepatocytes, mainly a shift to GM3 from GM2, might exert a suppressive effect on lipogenesis by attenuating levels of insulin signaling (Fig 8).

## Discussion

Until now, intriguing evidence has been reported that GSLs play an important role in the regulation of liver metabolic status. Pharmacologic inhibition of glucosylceramide synthase, an enzyme catalyzing the synthesis of precursors of the majority of more complex GSLs such as gangliosides, ameliorated hepatosteatosis in ob/ob mice [19] and HFD-fed mice [27]. Conversely, hepatic overexpression of *NEU3*, a key enzyme for ganglioside hydrolysis, increased hepatic lipid accumulation in mice receiving a standard diet and HFD [28]. These findings suggest that specific GSL species or combinations of some of them might play pivotal regulatory roles in hepatic lipid accumulation. Nonetheless, it is not clear which GSL structure is responsible for the observed effects, and it is difficult to distinguish the effects of a particular ganglioside among multiple GSLs present. Here, we reported, for the first time, that DKO mice of GM2S and GD3S, which lack all gangliosides except GM3, were resistant to HFD-induced hepatosteatosis. Furthermore, gangliosides in the livers of DKO mice shifted to GM3, while WT livers mainly expressed GM2. These findings suggest that GM3 might act as an apparent negative regulator of hepatosteatosis *in vivo*.

Over the past two decades, findings that GM3 negatively regulate insulin signaling have been accumulating [14, 15]. However, the functional significance of GM3 in the regulation of insulin signaling has not yet been fully elucidated in liver or hepatocytes, while it is one of the major insulin-sensitive and lipogenic organs. Also, it is controversial whether insulin signaling diminished by GM3 actually aggravates the pathological conditions in metabolic disorders. In this study, we clearly demonstrated that hepatic lipogenesis-related molecules including insulin signaling components were down-regulated in HFD-fed DKO mice which escaped hepatosteatosis. Also, when hepatocytes were cultured in serum plus medium, the expressions and/or activations in the downstream components of insulin signaling were reduced in DKO hepatocytes compared with WT hepatocytes, leading to lower levels of lipogenic molecules. These facts obtained from our *in vitro* and *in vivo* studies suggest that GM3 attenuate insulin signaling for lipogenesis, at least in mouse hepatocytes, providing a protection mechanism of HFD-induced hepatosteatosis. A recent report demonstrated that the absence of GM3 enhanced insulin signaling and adipogenesis in adipocyte-like cells [17]. In other words, GM3 might negatively modulate insulin signaling to reduce lipogenic actions in lipogenic cells.

The signaling pathway transduced from IR to mTOR-SREBP-1c is required for the stimulation of lipogenic gene transcription in liver [24, 29]. Our study confirmed that insulin stimulation led to marked up-regulation of FASN, one of the targets of SREBP-1c [26], in WT hepatocytes. On the other hand, induction of FASN by insulin in DKO hepatocytes was minimal, leading to significantly decreased FASN than in WT hepatocytes. It was reported that FAT/CD36, which does not possess SREBP-1c binding element, is induced by insulin via Akt phosphorylation [30]. Likewise, FAT/CD36 was slightly, but significantly, enhanced by insulin regardless of the genotype in hepatocytes. Nonetheless, the induced levels of FAT/CD36 were reduced in DKO hepatocytes. Unfortunately, insulin stimulation failed to up-regulate ACC, another target of SREBP-1c, in hepatocytes of either genotype, whereas SREBP-1c is primarily involved in the regulation of genes related to fatty acid and TG synthesis including ACC and FASN [26]. It is well known that AMPK activation is linked with a host of metabolic improvements, then activated AMPK inhibits transcriptions and/or activations of SREBP-1c and lipogenic molecules including ACC, leading to decrease lipogenesis [31, 32]. Although insulin reduced phosphorylation of AMPK in mouse hepatocytes, phosphorylation ratios of AMPK were higher in DKO hepatocytes than WT before and after insulin stimulation (at 60 min). These results might cause the reduced ACC in DKO hepatocytes to diminish lipogenesis in hepatocytes expressing GM3. As shown in Fig 8, diminished expressions and/or insulin-mediated activations of signaling components which resulted in lower induction of lipogenic molecules in hepatocytes might explain why DKO mice were resistant to HFD-induced hepatosteatosis. Consequently, the modified gangliosides, i.e., a shift to GM3 from GM2, might be essential for the maintenance of healthy status and functions at least in mouse hepatocytes. On the other hand, activations of insulin signal cascade in serum starved condition were not significantly different between WT and DKO hepatocytes, except decreased levels of phosphorylated forms of IR at 5 min after insulin stimulation. Therefore, attenuated effects on downstream signaling of IR phosphorylation by GM3 might need support of basal physiological condition with serum.

Mechanisms by which lipogenic insulin signaling is attenuated in DKO hepatocytes remain to be elucidated. Generally, GSLs regulate cellular signaling pathways by interacting with components of the signal transduction machinery in membrane microdomains, lipid rafts [33], or glycolipid-enriched microdomains (GEM/rafts) [34, 35]. There have been a number of reports on dynamic quantitative and qualitative changes in GEM/raft components accompanied by ganglioside modifications *in vitro* [36–38] and *in vivo* [9, 10]. Since DKO mice displayed the dispersion of raft-residing molecules including caveolin-1 from GEM/rafts in cerebellum [9, 10], insulin signaling might be attenuated due to disturbed IR localization from GEM/rafts in DKO hepatocytes. Although it was reported that manipulation of the GEM/raft (i.e., cholesterol depletion by cyclodextrin or GSL clustering by antibody) modulated IR signaling in human hepatoma cell line HuH7 [39], there is little direct evidence concerning the association between insulin signaling and GEM/rafts in normal hepatocytes. The mechanisms by which ganglioside changes affect insulin signaling via GEM/rafts in hepatocytes remain to be clarified in the future.

Since it has been suggested that GM3 is involved in the pathophysiological changes associated with obesity and/or metabolic disorders by inhibiting insulin signaling [14, 15], we originally expected that DKO mice, which were unable to produce all gangliosides except GM3 systemically, would exhibit severe metabolic disorders due to insulin dysfunction. Surprisingly, DKO mice aged at 20 weeks old did not show disfunctions in control of blood glucose during GTT or ITT, and older DKO mice aged at 35–40 weeks old maintained glucose tolerance though age-matched WT showed glucose intolerance. Furthermore, fasting blood glucose levels were significantly lower in DKO mice than WT mice with both aging and HFD loading. These results reveal that only GM3 expression does not necessarily result in systemic insulin resistance, and rather GM3 might be important for maintenance of glucose homeostasis. Because coordination of circulating glucose by insulin is the result of a complex regulatory system involved in various tissues such as skeletal muscles, adipose tissues, and liver, diminished insulin signaling in DKO hepatocytes might have minor impacts on systemic metabolic homeostasis. Also, DKO mice escaped age- or HFD-induced obesity notwithstanding similar energy intake and expenditure to WT mice. Though those regulating mechanisms should be elucidated in future studies, our present findings strongly indicate that GM3 might not necessarily be a pathogenic factor inducing obesity and obesity-related metabolic diseases including hyperglycemia.

Whereas exclusion of GM3 may be an effective strategy to study roles of GM3 in tissues or cells where GM3 is mainly expressed, it should be carefully considered whether remaining and/or eliminated GSLs along with GM3 loss might be involved in changes in the sensitivity to insulin. In fact, many researchers have reported that suppression of GM3 expression resulted in enhancement of insulin responses [14–20]. However, these findings are insufficient to understand accurate and direct outcomes of GM3 expression due to its substitution into other GSL structures. Contrarily, DKO mice of GM2S and GD3S are very valuable to assess the effects of GM3 without influence of other gangliosides, because gangliosides expressed in DKO mice are simply replaced with GM3 through a whole-body. All our findings directly prove that expression of GM3 attenuates insulin signaling in mouse liver or hepatocytes.

## Supporting information

S1 Table Primer sequences used for real-time RT-PCR. TNFα, tumor necrosis factor α; MCP1, monocyte chemotactic protein 1; IL-6, interleukin-6; FAT/CD36, fatty acid translocase; FABP1, fatty acid binding protein 1; Mttp, microsomal triglyceride transfer protein; Dgat2, diacylglycerol O-acyltransferase 2; ApoB, apolipoprotein B; Fasn, fatty acid synthase; SREBP-1c, sterol regulatory element-binding protein-1c; PPAR gamma, peroxisome proliferator-activated receptor gamma; GAPDH, glyceraldehyde-3-phosphate dehydrogenase.
(TIF)

S1 Fig No marked alteration in the metabolic phenotypes of DKO mice lacking GM2S and GD3S. (**A**) Metabolic measurement was performed using an Oxymax laboratory animal monitoring system (Columbus Instruments, Columbus, OH, USA). Mice at 20 weeks of age were individually housed in a four-chamber with a 12-h light / 12-h dark cycle in an ambient temperature of 22–24°C. The oxygen consumption (VO_2_), respiratory exchange ratio (RER), and energy expenditure (EE) of individual mice were measured every 5 min for 24 h. During this study, mice had *ad libitum* access to food and water. Data are expressed as means ± S.D. (**B**) Metabolic measurement was performed in mice at 35–40 weeks of age Data are expressed as means ± S.D. (**C**) Food consumption was measured during one week to calculate average energy intake per day. Open and closed bars show WT and DKO mice, respectively. Data are expressed as means ± S.D.
(TIF)

S2 Fig DKO mice attenuated HFD-induced adiposity without reduced energy intake. (**A**) Food consumption was measured every week to calculate average amounts of food intake per day. *P<0.05 as compared between WT and DKO mice. ^###^P<0.001 as compared between CD and HFD groups. (**B**) Tissue weights of epididymal fat were measured after the end of the 15-week HFD loading experiment. Open and closed bars show WT and DKO mice, respectively. Data are expressed as means ± S.D. *P<0.05 and **P<0.01 as compared between WT and DKO mice. ^###^P<0.001 as compared between CD and HFD groups. (**C**) Hematoxylin and eosin staining of epididymal fat tissues of CD-fed or HFD-fed mice. (**D**) Quantification of adipocyte sizes of epididymal fat was performed using ImageJ-4 for randomly choosing image, and average sizes were calculated. Open and closed bars indicate WT and DKO mice, respectively. Data are expressed as means ± S.D. ***P<0.001 as compared between WT and DKO mice. ^###^P<0.001 as compared between CD and HFD groups.
(TIF)

S3 Fig Expression levels of signaling molecules after insulin stimulation in primary hepatocytes cultured in medium without serum. (**A**) Expression levels of insulin signaling molecules in hepatocytes were analyzed by immunoblotting. (**B**) Relative expression levels of phosphorylated forms and total amounts by measuring band intensities after correction with those of β-Actin, and (**C**) phosphorylation levels estimated as ratios of phosphorylated or cleaved forms to total amounts. The numbers of mice examined were WT, n = 2; DKO, n = 3. Data are expressed as means ± S.D. *P<0.05 and ***P<0.001 as compared between WT and DKO hepatocytes. Open and closed bars indicate WT and DKO hepatocytes, respectively. ^#^P<0.05, ^##^P<0.01 and ^###^P<0.001as compared between basal and insulin stimulation.
(TIF)

S4 Fig Insulin-stimulated activation of signaling molecules in primary hepatocytes. Phosphorylation or activation levels of signaling molecules are estimated as ratios of phosphorylated or cleaved forms to total amounts presented in Fig 6A. Open and closed bars indicate WT and DKO hepatocytes, respectively. *P<0.05 as compared between WT and DKO hepatocytes. ^#^P<0.05 and ^##^P<0.01 as compared between basal and insulin stimulation.
(TIF)

S1 Raw images (PDF)
