# Supplementary figures and images for: Ganglioside GM3 prevents high fat diet-induced hepatosteatosis via attenuated insulin signaling pathway
Source: PLoS One. 2023 Feb 24;18(2):e0281414. doi: 10.1371/journal.pone.0281414 (PMC9956598; doi:10.1371/journal.pone.0281414)

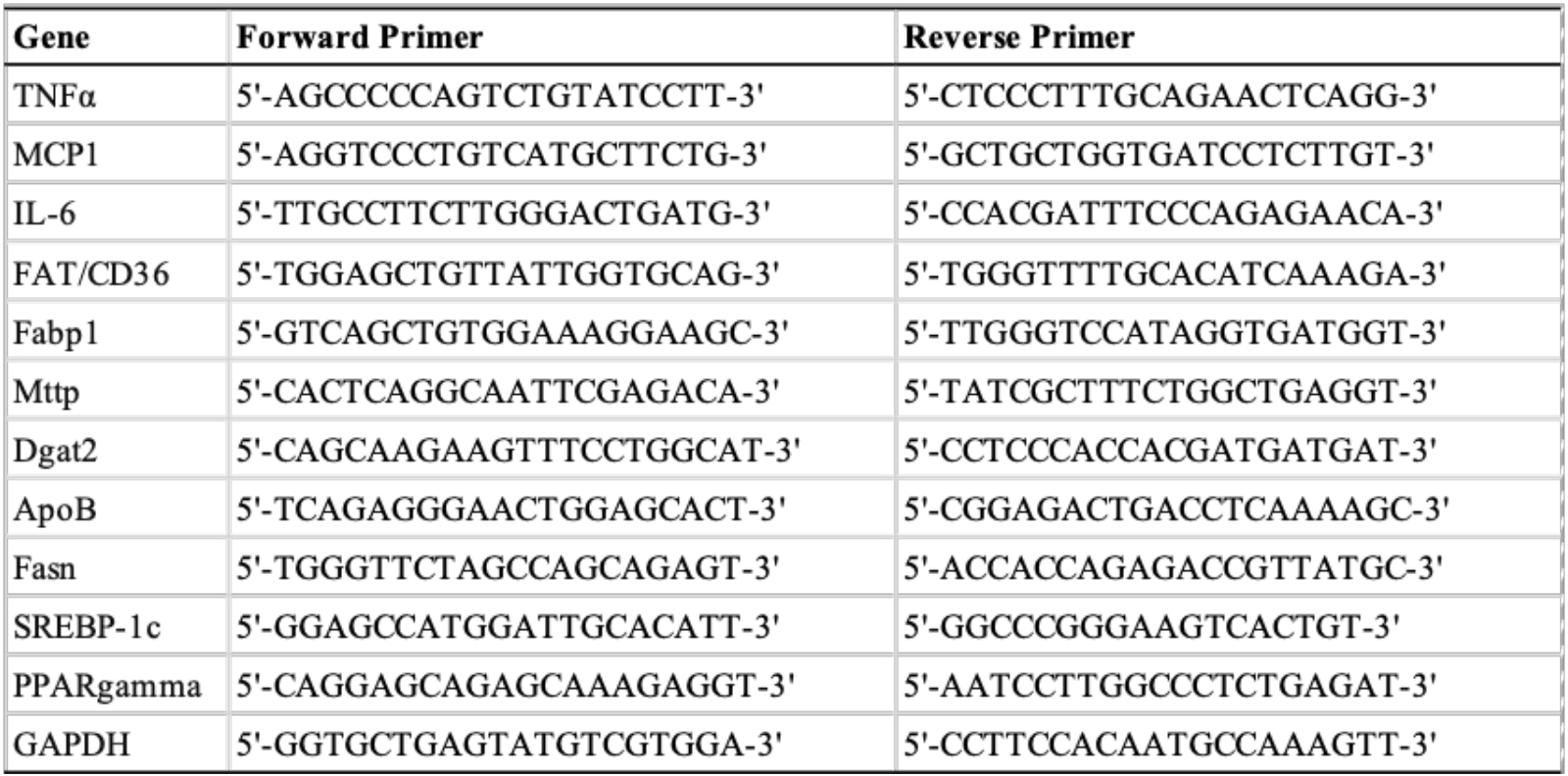

Supplement: S1 Table — TNFα, tumor necrosis factor α; MCP1, monocyte chemotactic protein 1; IL-6, interleukin-6; FAT/CD36, fatty acid translocase; FABP1, fatty acid binding protein 1; Mttp, microsomal triglyceride transfer protein; Dgat2, diacylglycerol O-acyltransferase 2; ApoB, apolipoprotein B; Fasn, fatty acid synthase; SREBP-1c, sterol regulatory element-binding protein-1c; PPAR gamma, peroxisome proliferator-activated receptor gamma; GAPDH, glyceraldehyde-3-phosphate dehydrogenase. (TIF) [file pone.0281414.s001.tif]

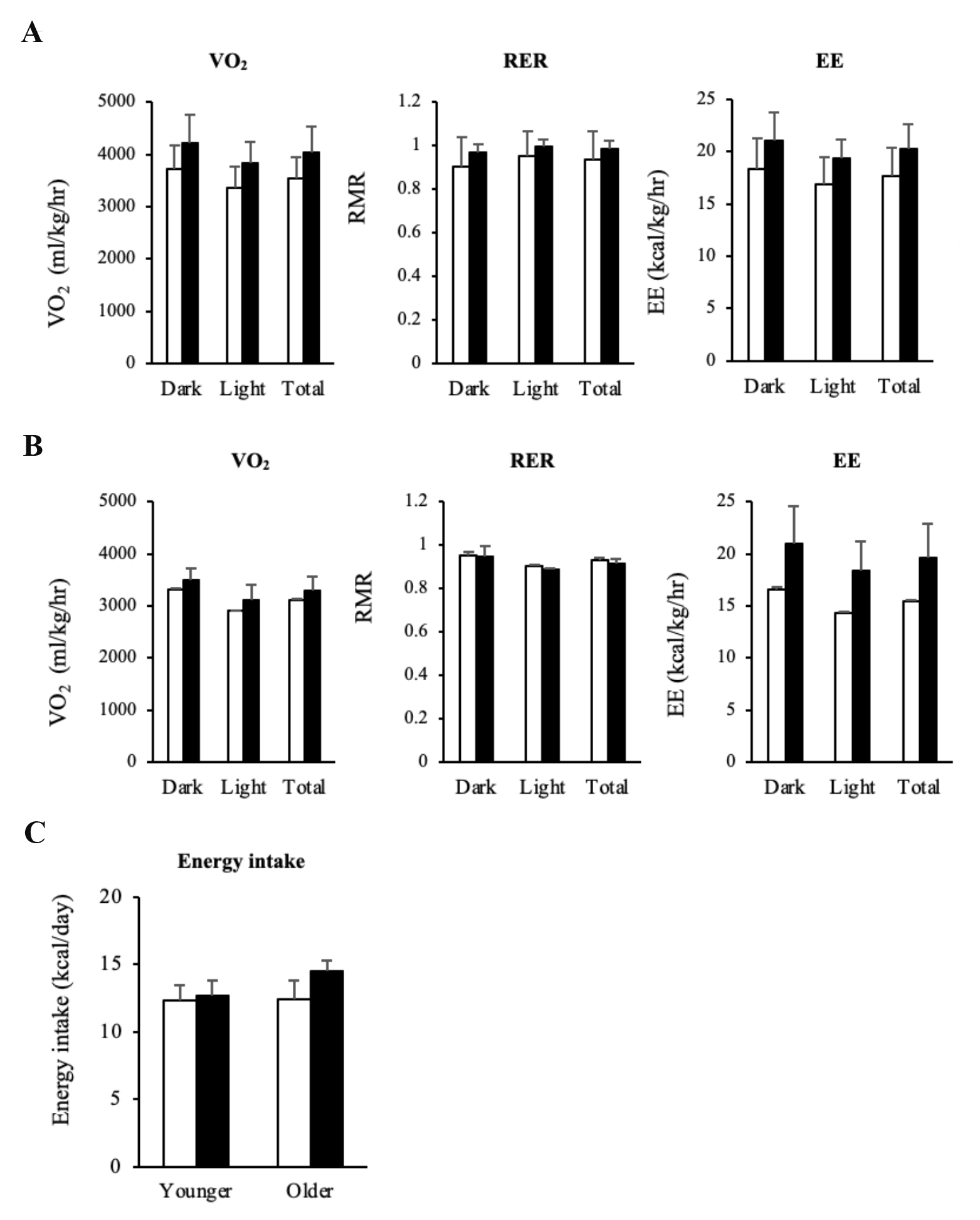

Supplement: S1 Fig — (A) Metabolic measurement was performed using an Oxymax laboratory animal monitoring system (Columbus Instruments, Columbus, OH, USA). Mice at 20 weeks of age were individually housed in a four-chamber with a 12-h light / 12-h dark cycle in an ambient temperature of 22–24°C. The oxygen consumption (VO2), respiratory exchange ratio (RER), and energy expenditure (EE) of individual mice were measured every 5 min for 24 h. During this study, mice had ad libitum access to food and water. Data are expressed as means ± S.D. (B) Metabolic measurement was performed in mice at 35–40 weeks of age Data are expressed as means ± S.D. (C) Food consumption was measured during one week to calculate average energy intake per day. Open and closed bars show WT and DKO mice, respectively. Data are expressed as means ± S.D. (TIF) [file pone.0281414.s002.tif]

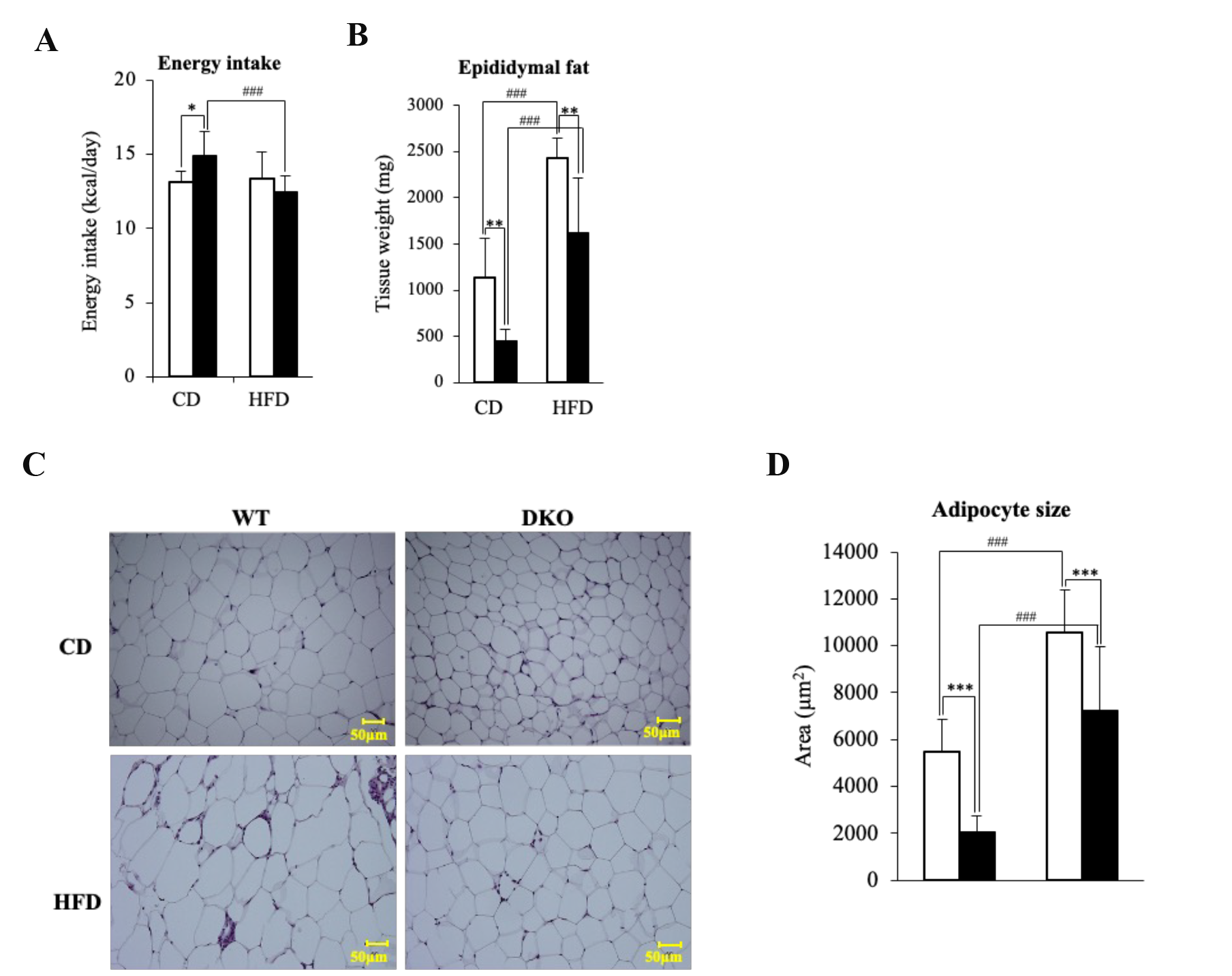

Supplement: S2 Fig — (A) Food consumption was measured every week to calculate average amounts of food intake per day. *P<0.05 as compared between WT and DKO mice. ###P<0.001 as compared between CD and HFD groups. (B) Tissue weights of epididymal fat were measured after the end of the 15-week HFD loading experiment. Open and closed bars show WT and DKO mice, respectively. Data are expressed as means ± S.D. *P<0.05 and **P<0.01 as compared between WT and DKO mice. ###P<0.001 as compared between CD and HFD groups. (C) Hematoxylin and eosin staining of epididymal fat tissues of CD-fed or HFD-fed mice. (D) Quantification of adipocyte sizes of epididymal fat was performed using ImageJ-4 for randomly choosing image, and average sizes were calculated. Open and closed bars indicate WT and DKO mice, respectively. Data are expressed as means ± S.D. ***P<0.001 as compared between WT and DKO mice. ###P<0.001 as compared between CD and HFD groups. (TIF) [file pone.0281414.s003.tif]

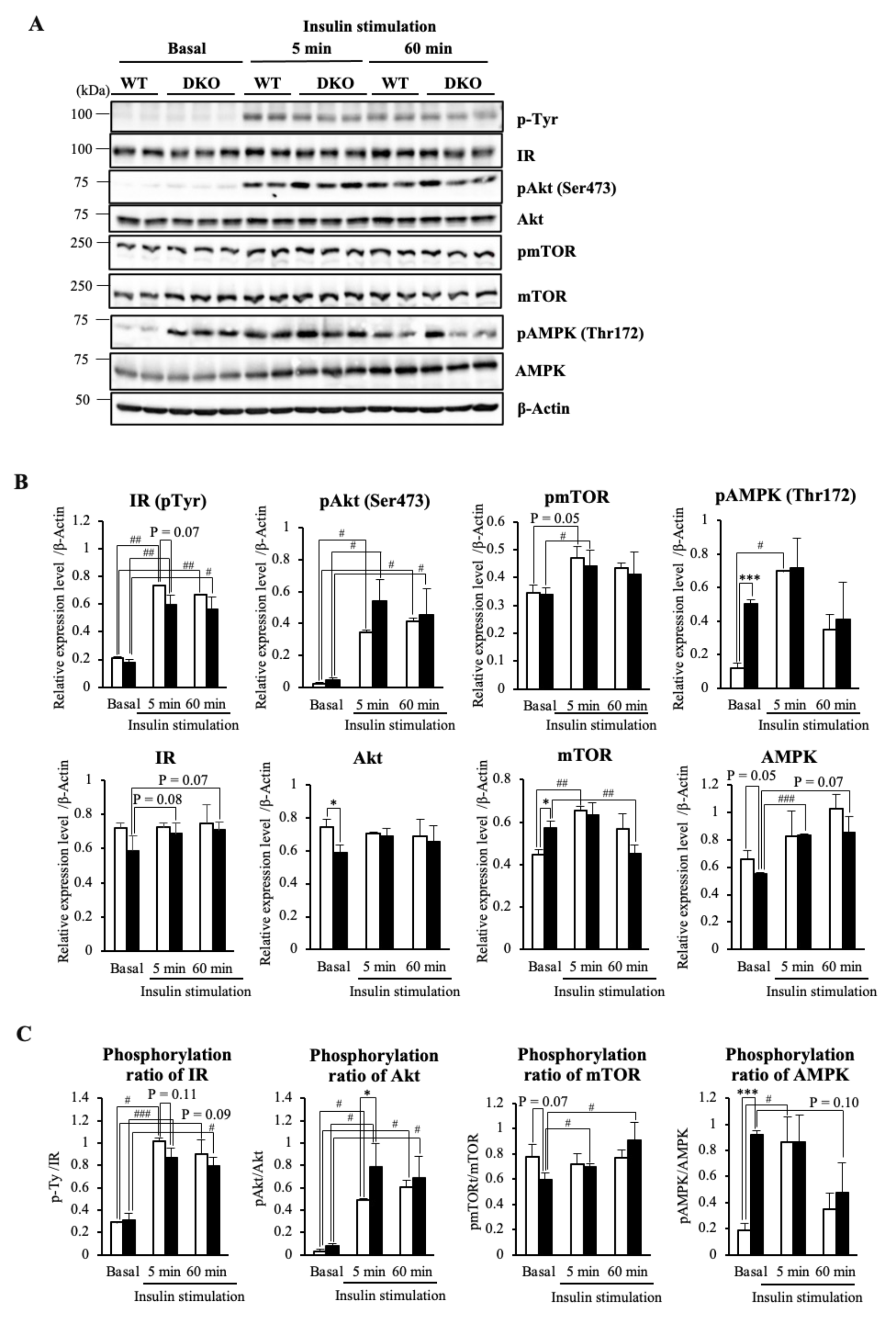

Supplement: S3 Fig — (A) Expression levels of insulin signaling molecules in hepatocytes were analyzed by immunoblotting. (B) Relative expression levels of phosphorylated forms and total amounts by measuring band intensities after correction with those of β-Actin, and (C) phosphorylation levels estimated as ratios of phosphorylated or cleaved forms to total amounts. The numbers of mice examined were WT, n = 2; DKO, n = 3. Data are expressed as means ± S.D. *P<0.05 and ***P<0.001 as compared between WT and DKO hepatocytes. Open and closed bars indicate WT and DKO hepatocytes, respectively. #P<0.05, ##P<0.01 and ###P<0.001as compared between basal and insulin stimulation. (TIF) [file pone.0281414.s004.tif]

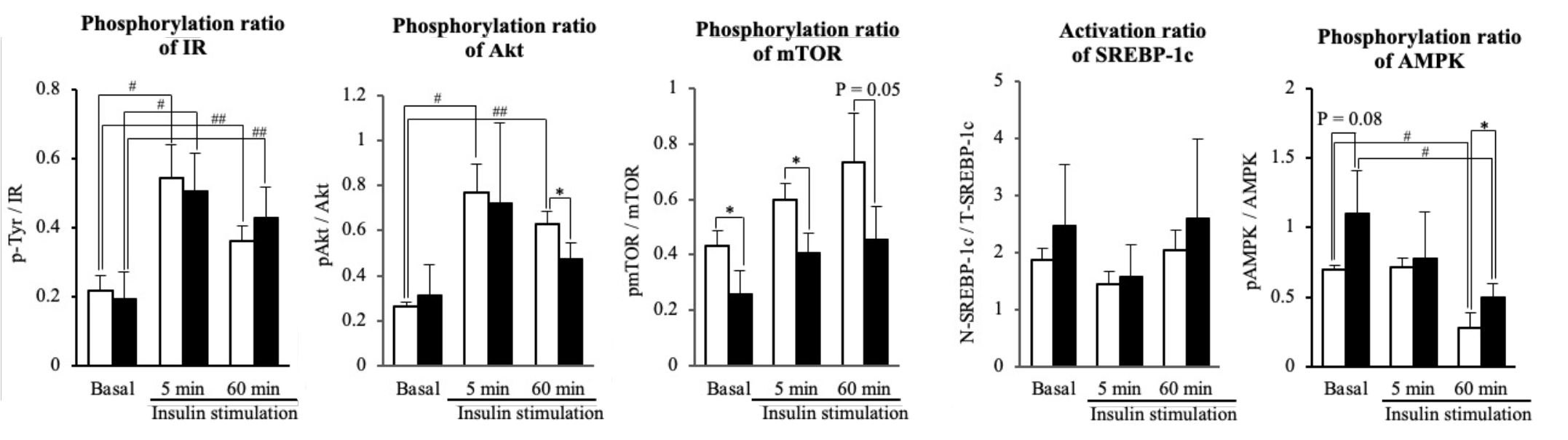

Supplement: S4 Fig — Phosphorylation or activation levels of signaling molecules are estimated as ratios of phosphorylated or cleaved forms to total amounts presented in Fig 6A. Open and closed bars indicate WT and DKO hepatocytes, respectively. *P<0.05 as compared between WT and DKO hepatocytes. #P<0.05 and ##P<0.01 as compared between basal and insulin stimulation. (TIF) [file pone.0281414.s005.tif]
