# Supplementary material for: Ganglioside GM3 prevents high fat diet-induced hepatosteatosis via attenuated insulin signaling pathway
Source: PLoS One. 2023 Feb 24;18(2):e0281414. doi: 10.1371/journal.pone.0281414 (PMC9956598; doi:10.1371/journal.pone.0281414)

Figure 4B:
FAT/CD36

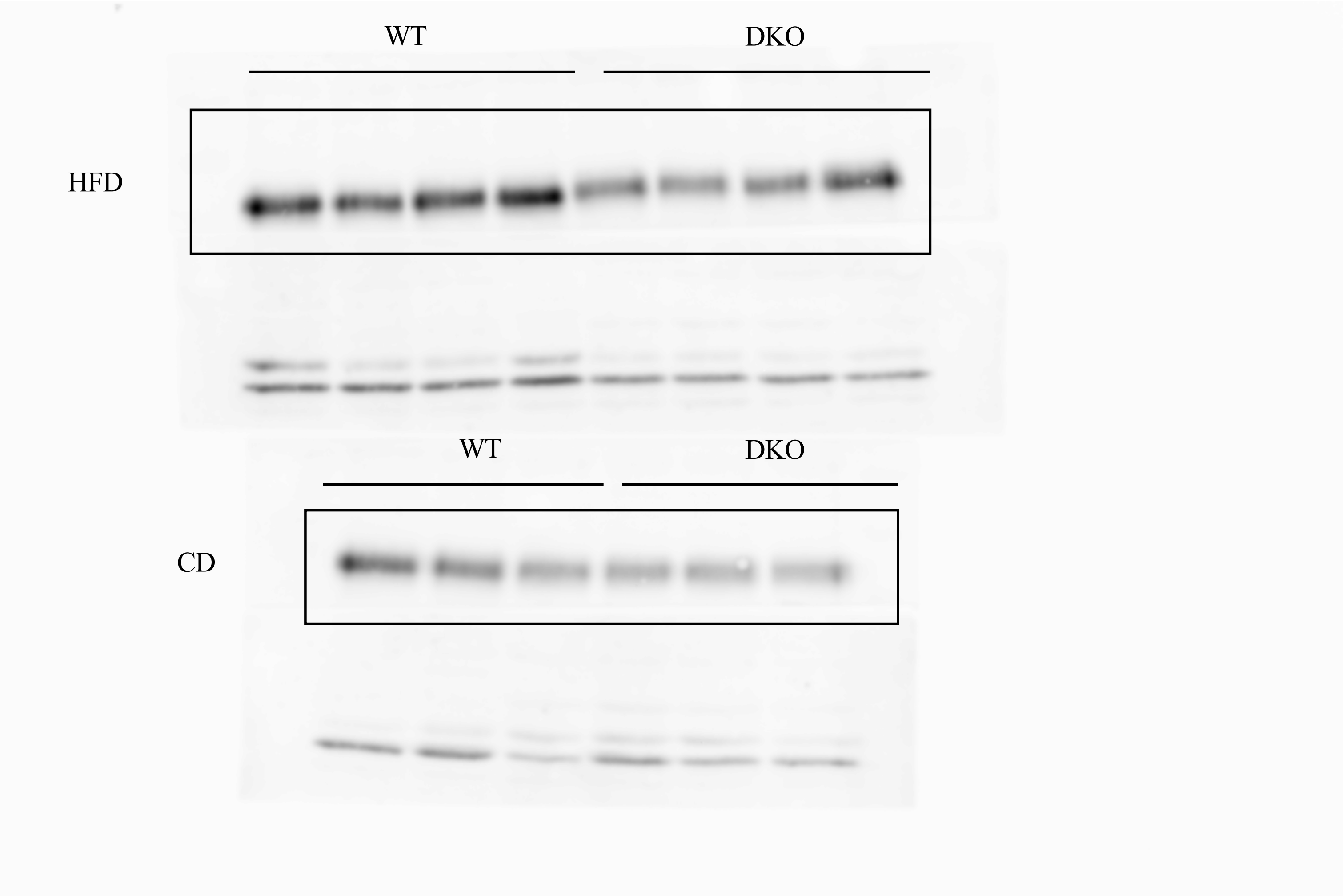

Figure 4B:
FABP1

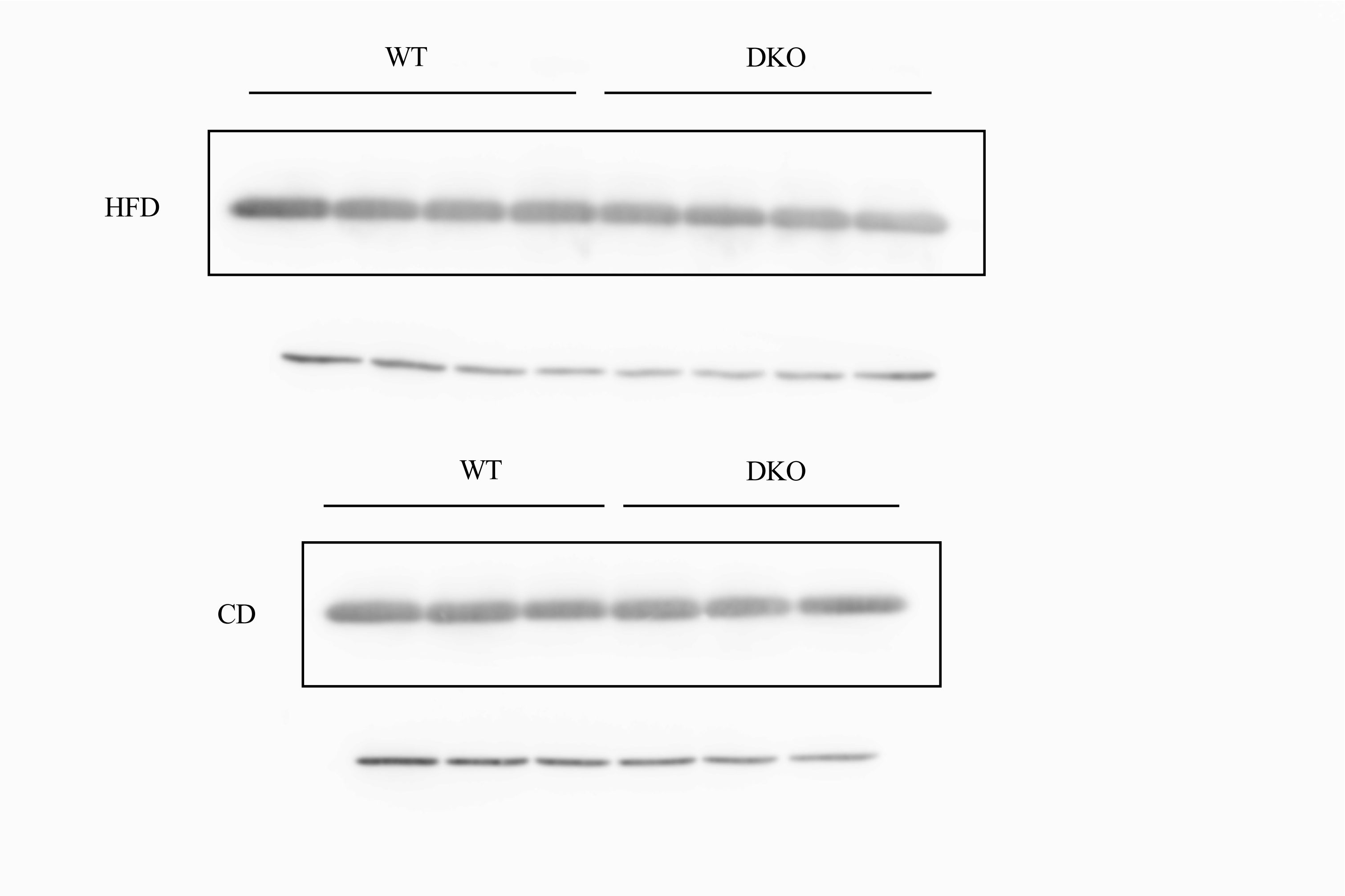

Figure 4B:
FASN

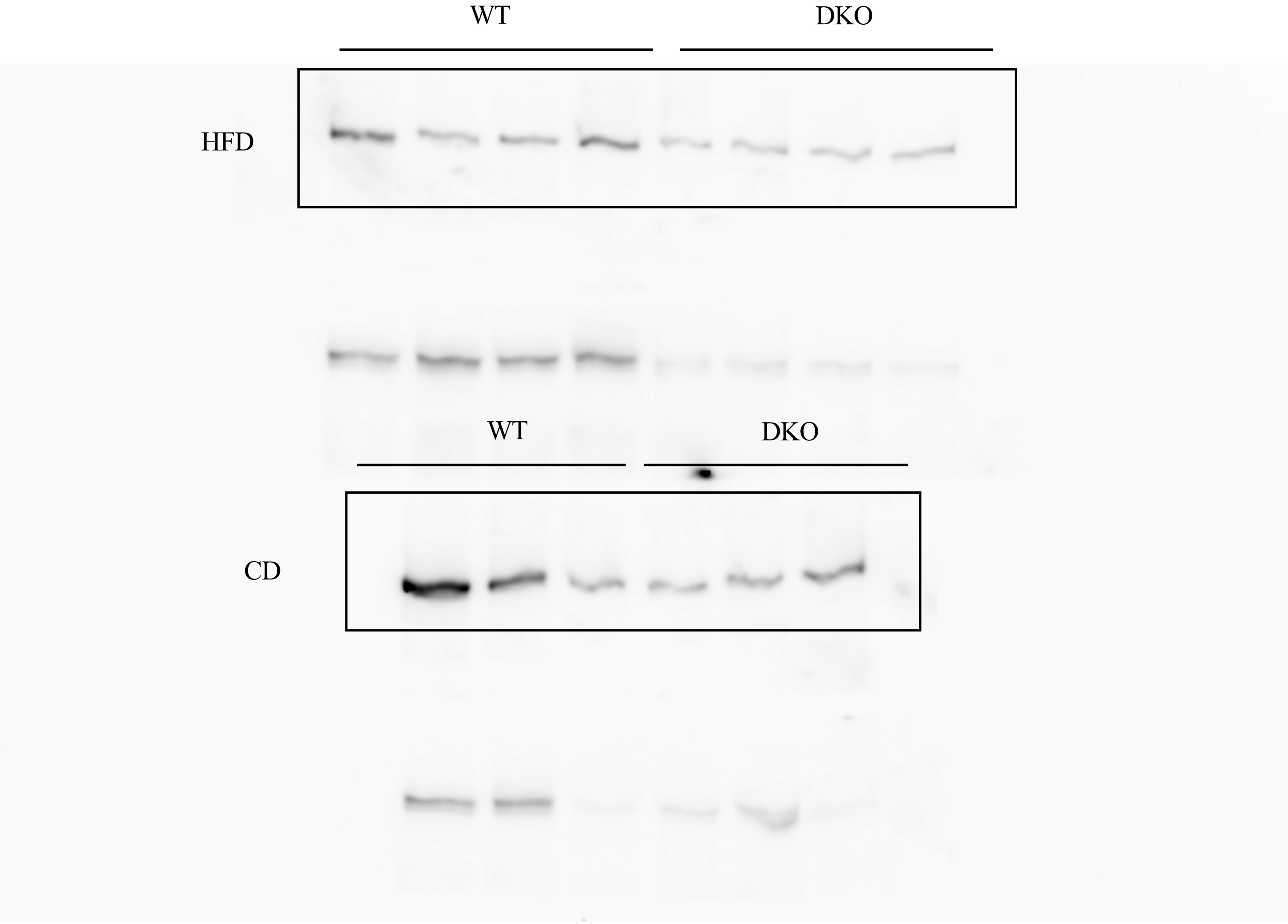

Figure 4B:
ACC

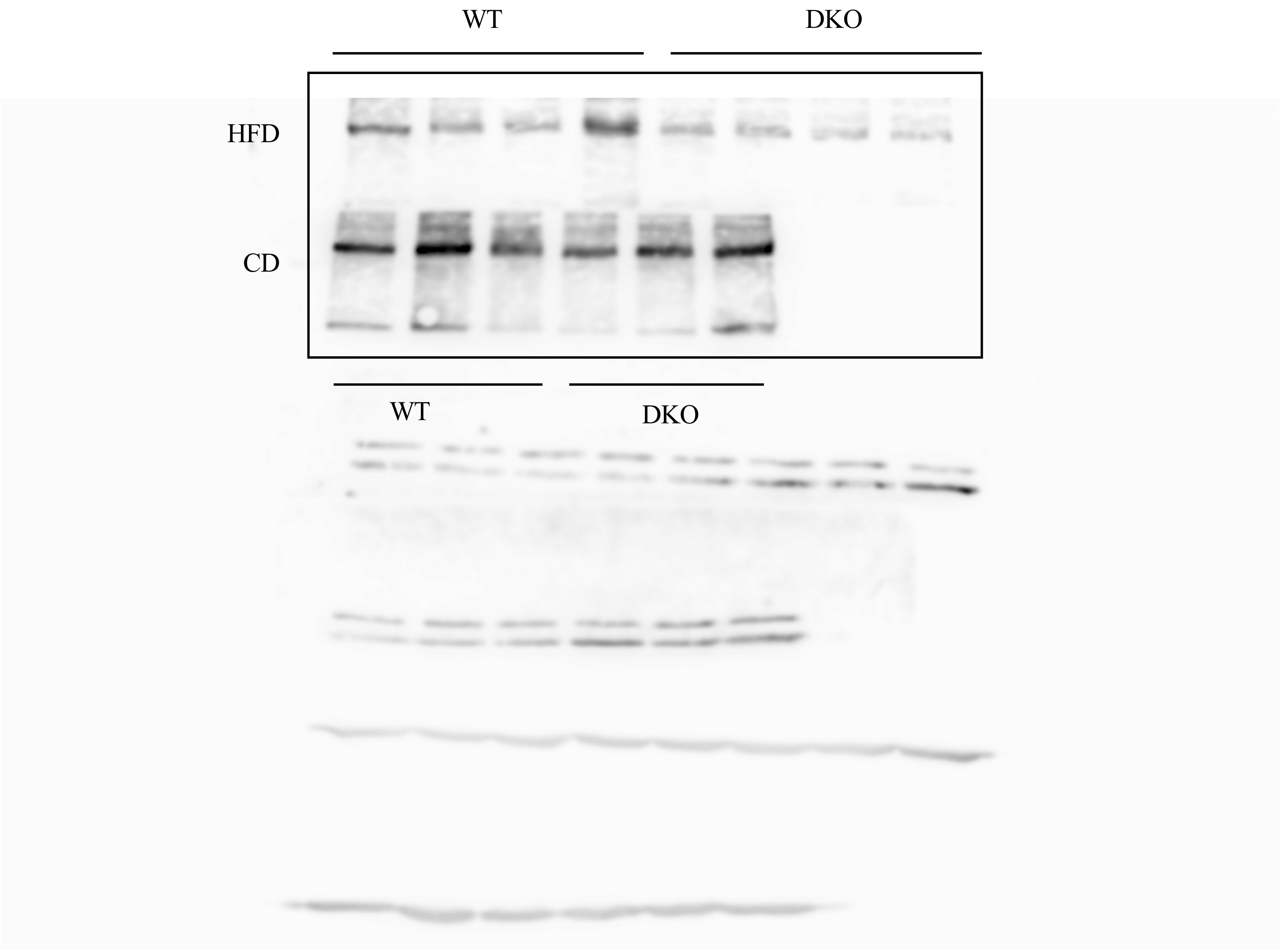

Figure 4B:
β—Actin

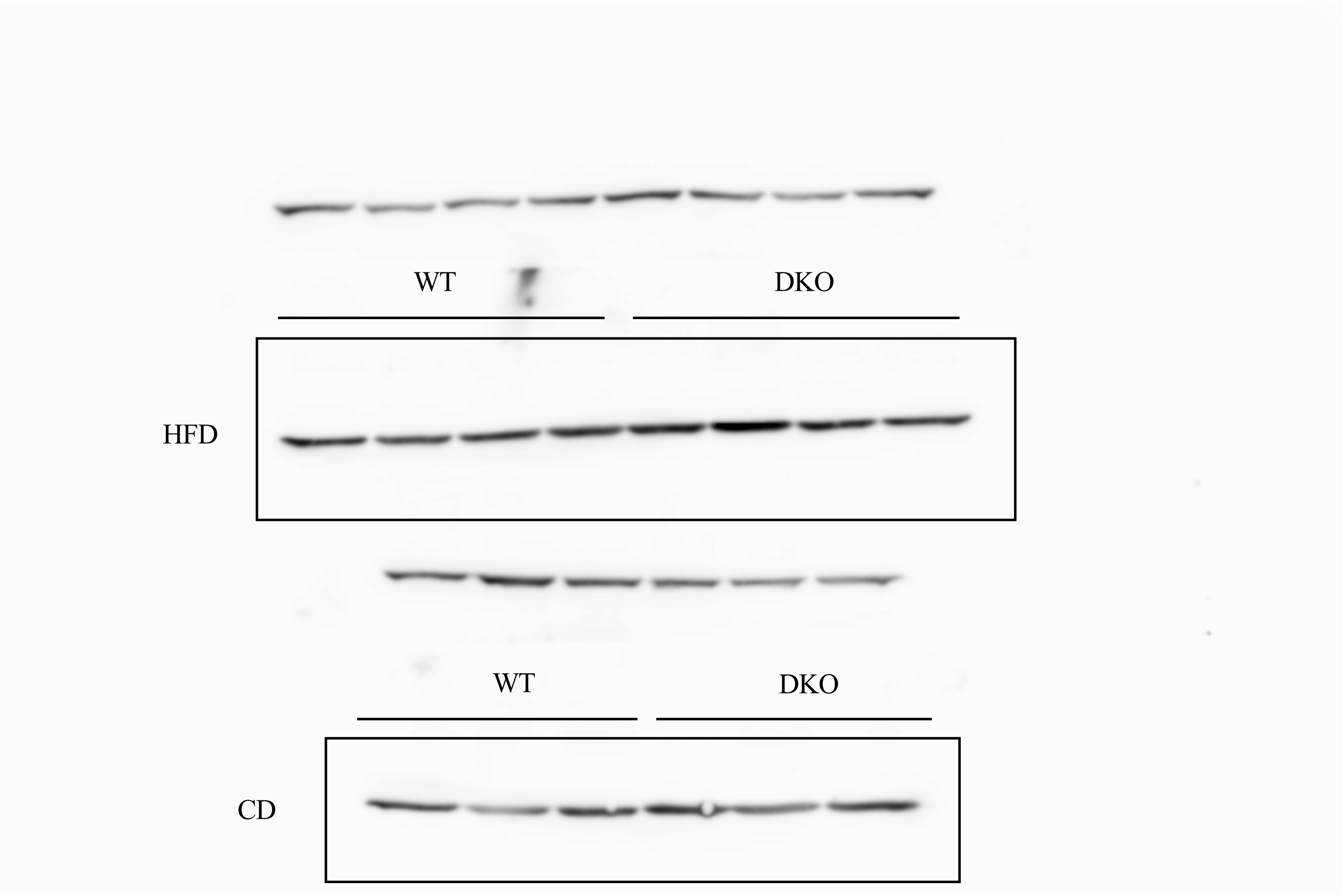

Figure 5A:
IR

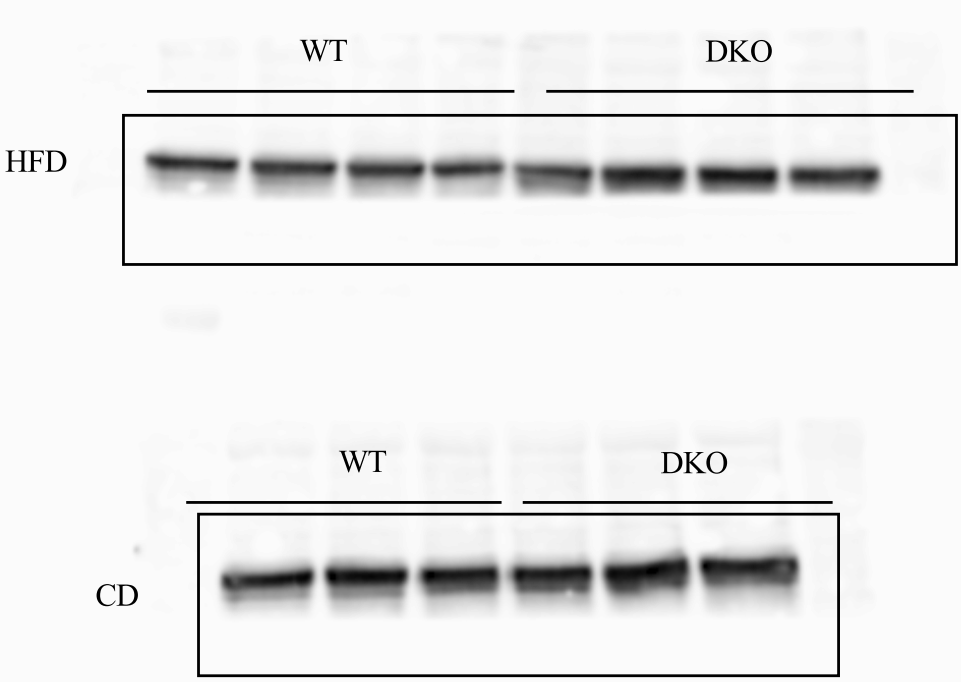

Figure 5A:
IRS-1

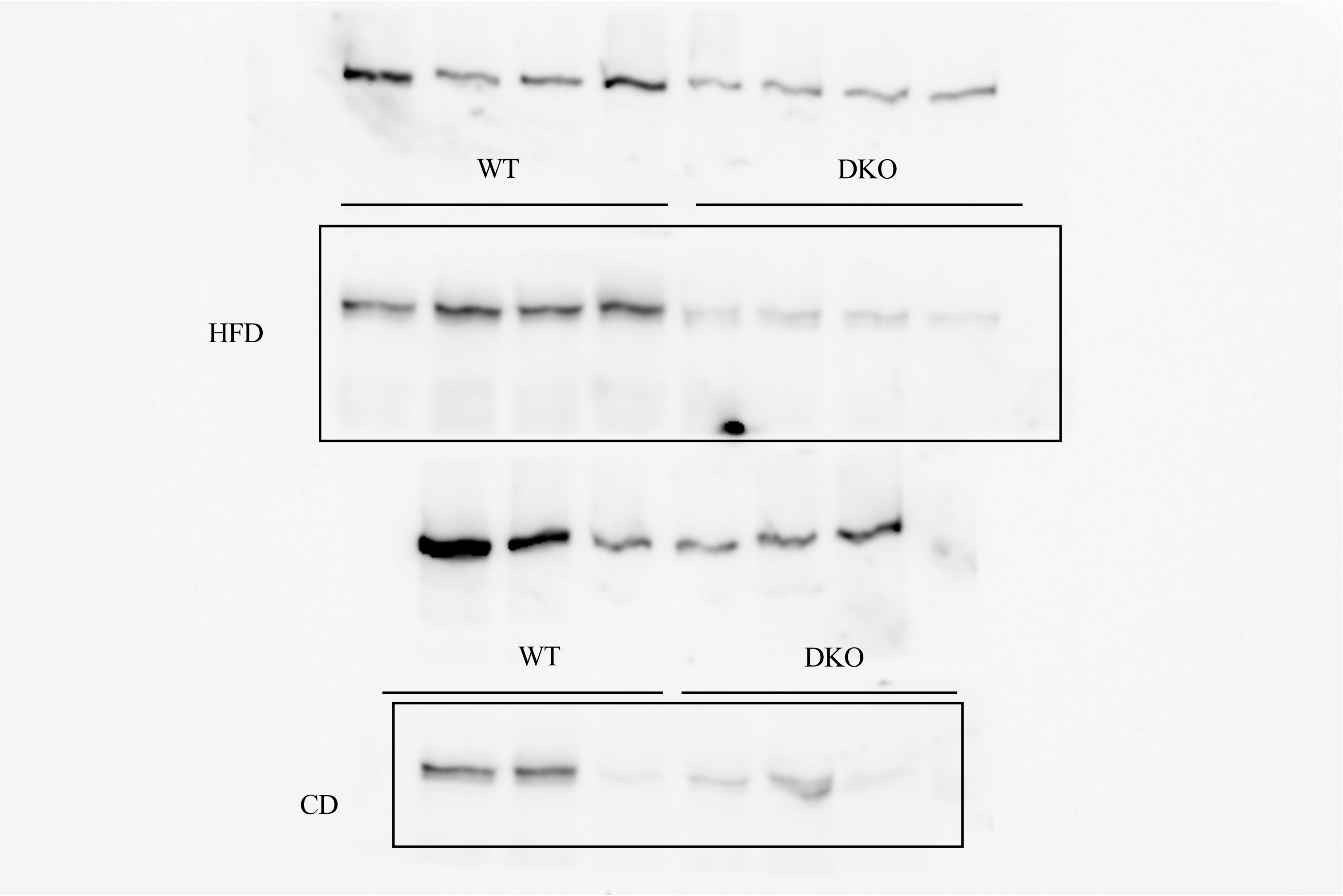

Figure 5A:
P!3-K

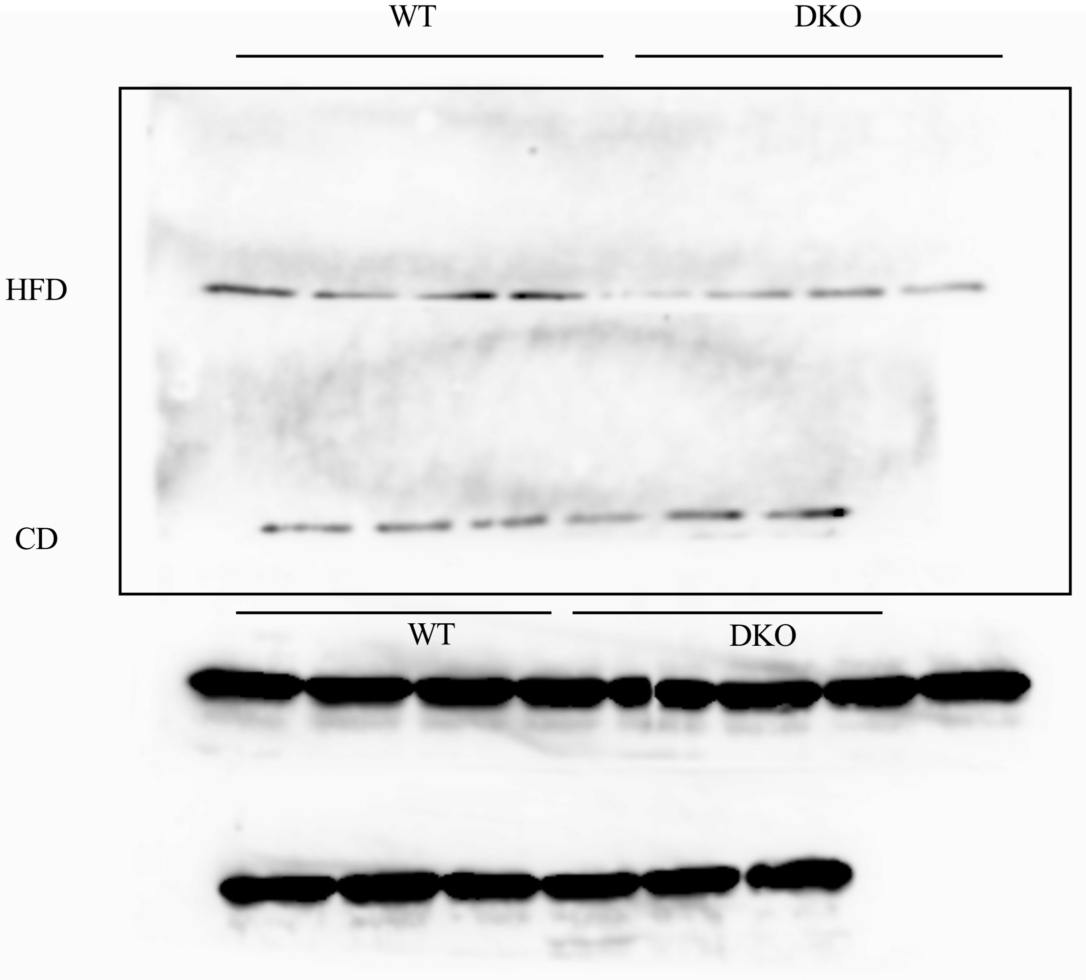

Figure 5A:
Akt

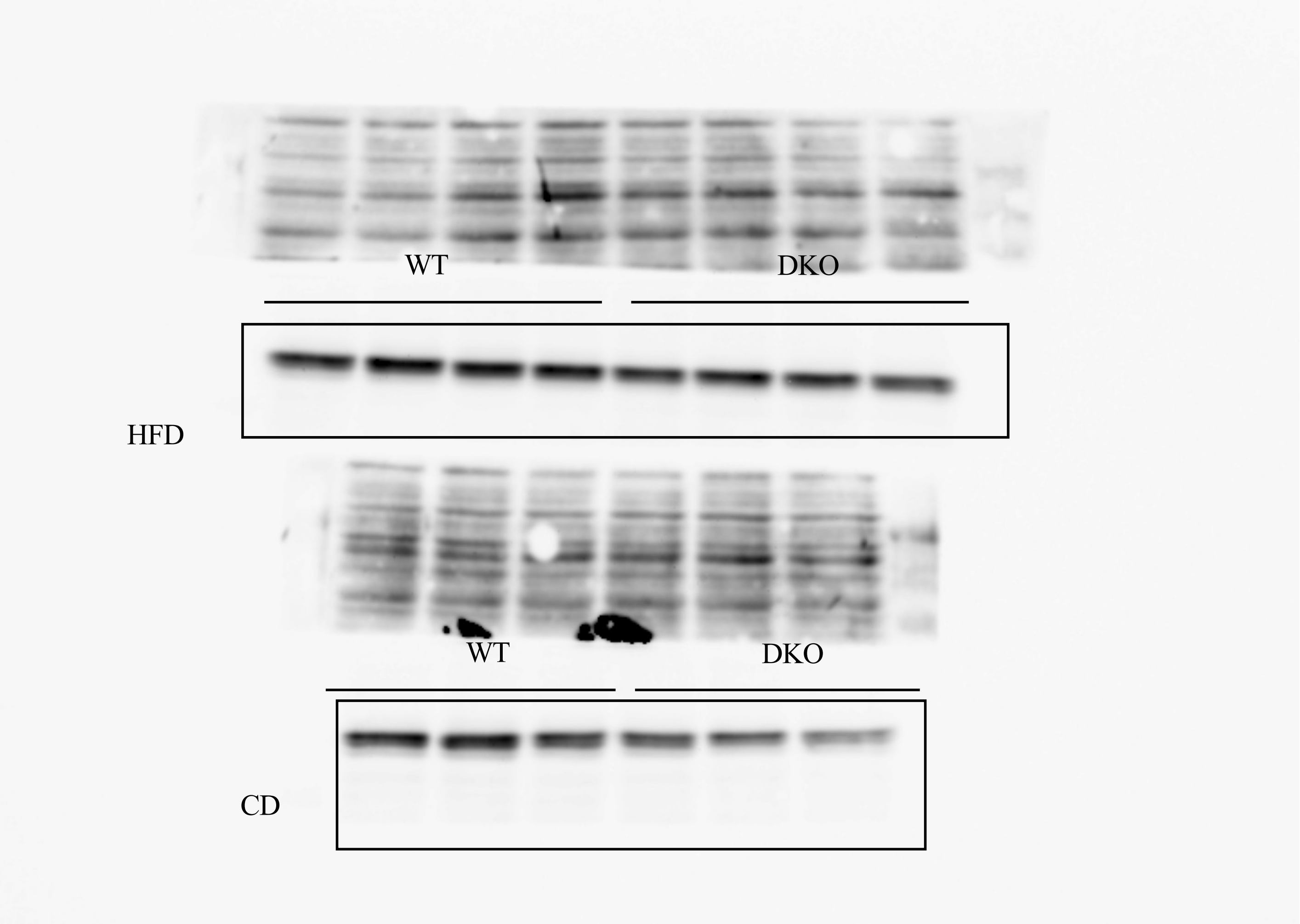

Figure 5A:
SREBP-1c

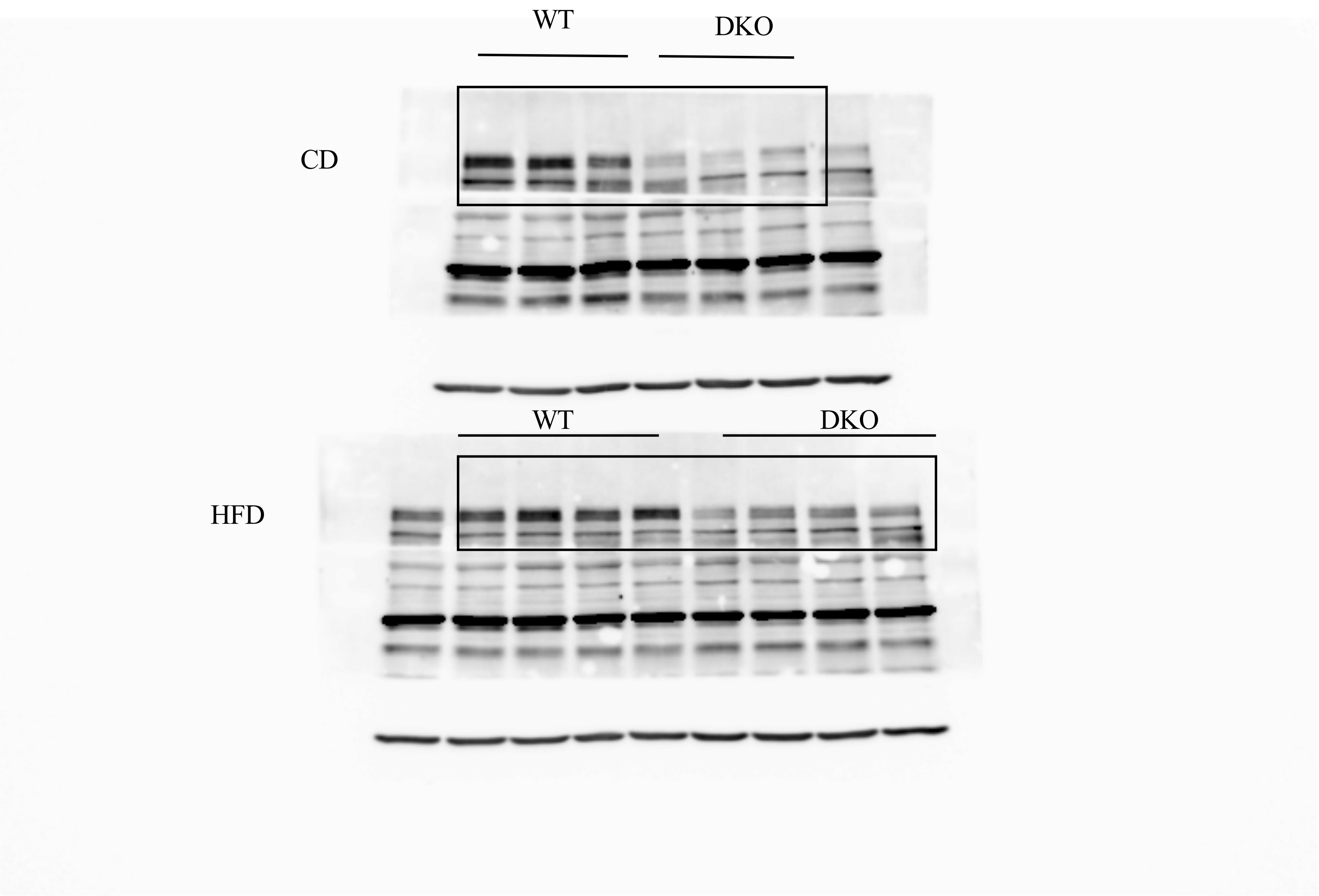

Figure 5A:
β-Actin

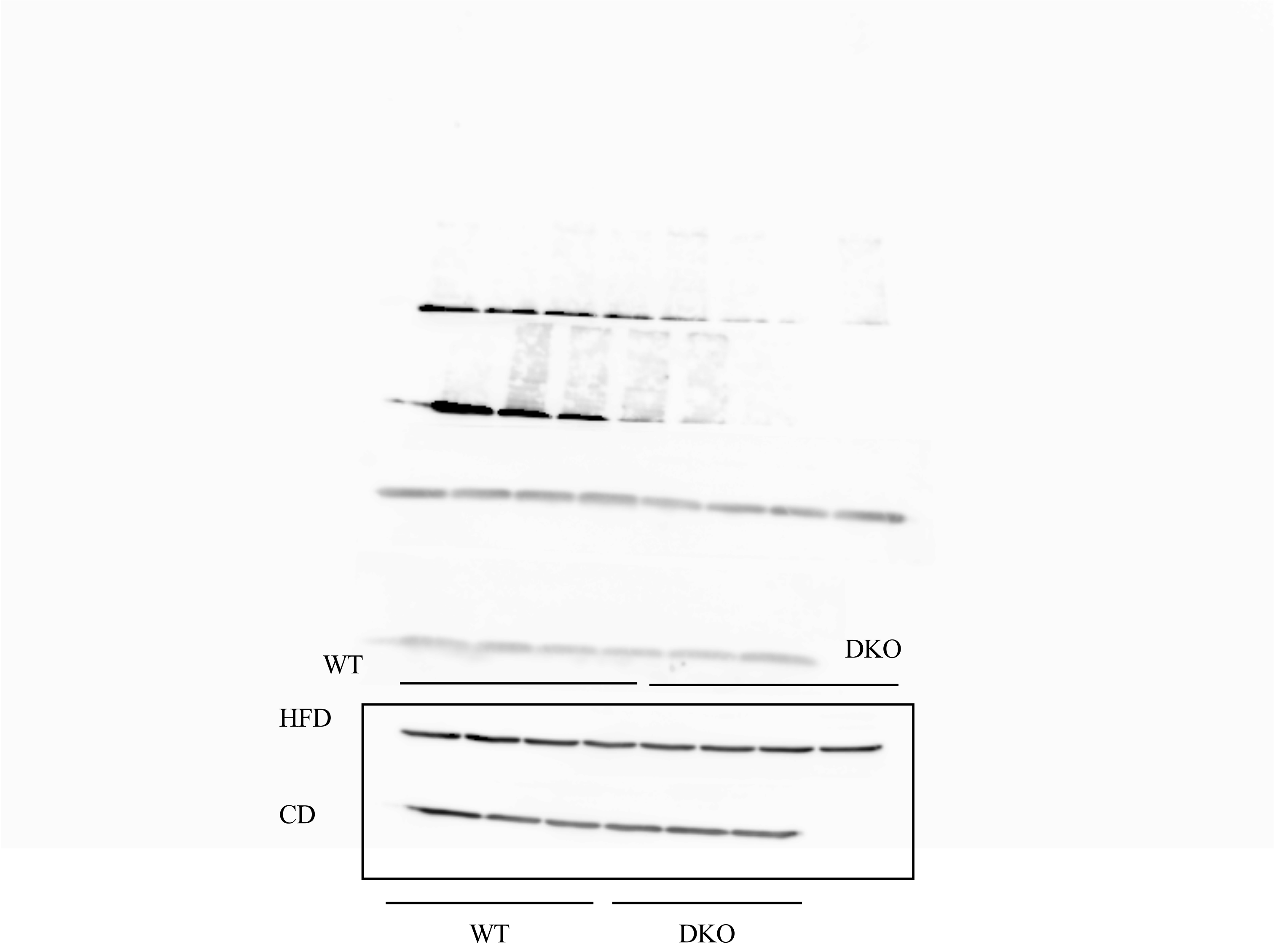

Figure 6A:
p-Tyr

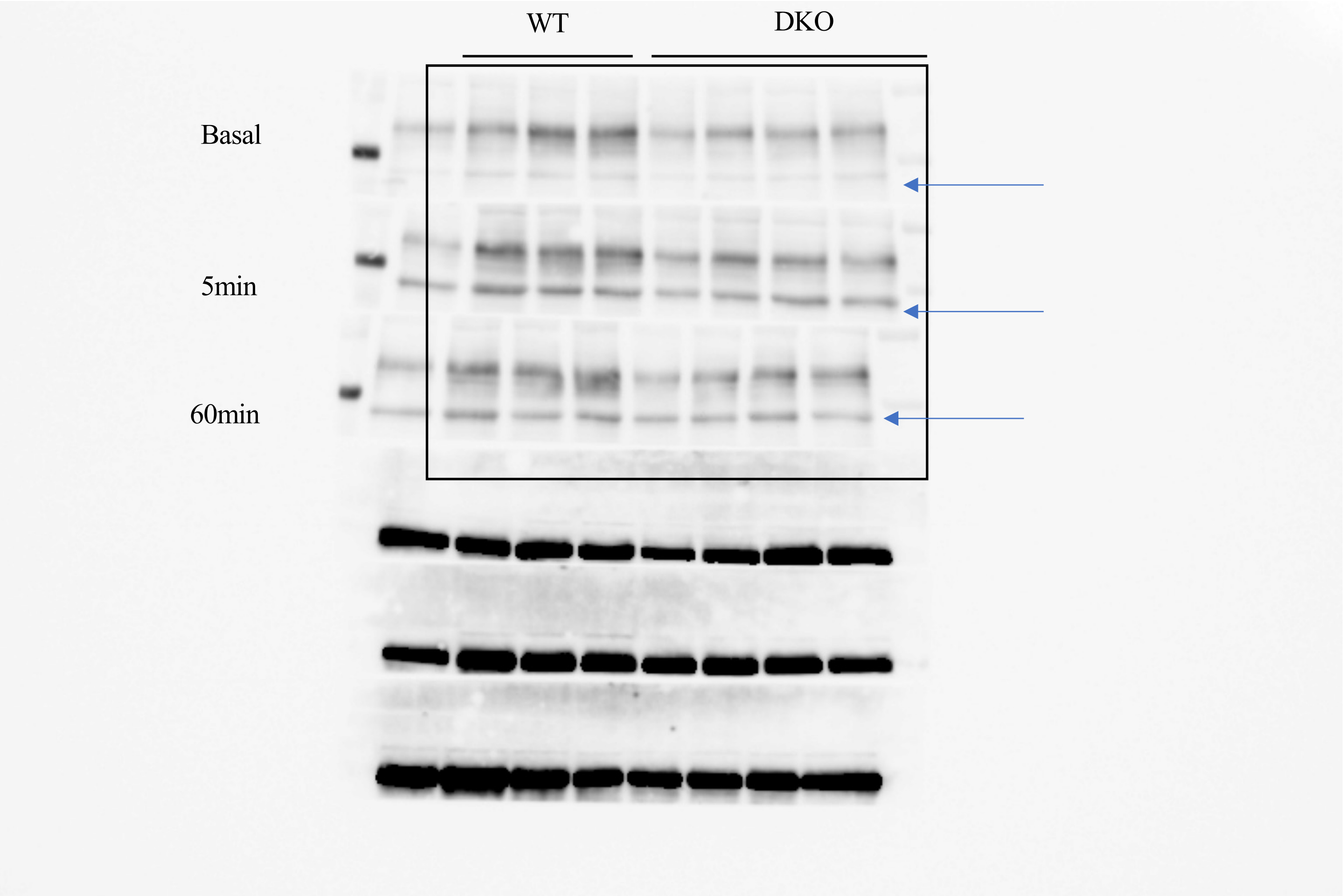

Figure 6A:
IR

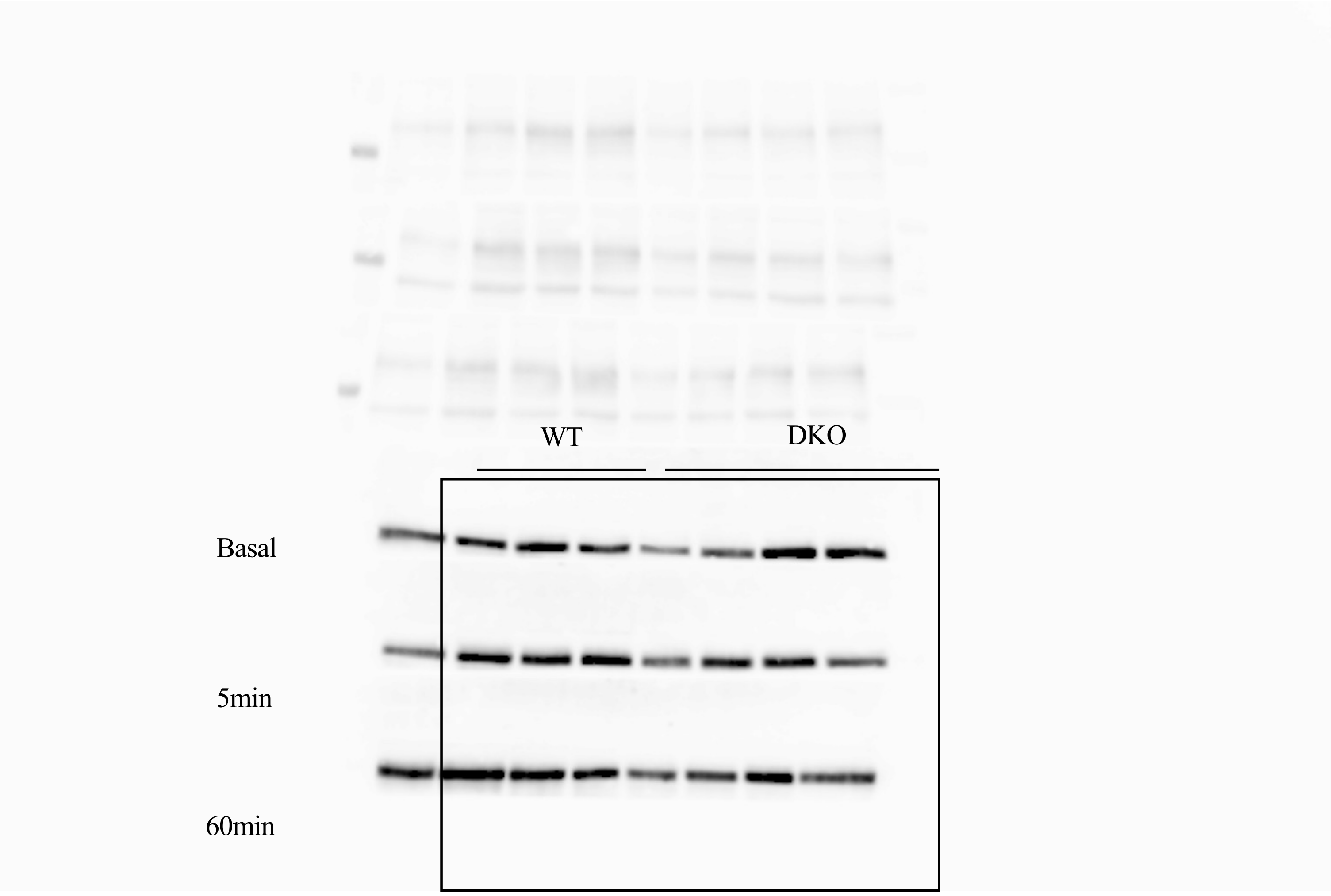

Figure 6A:
pAkt

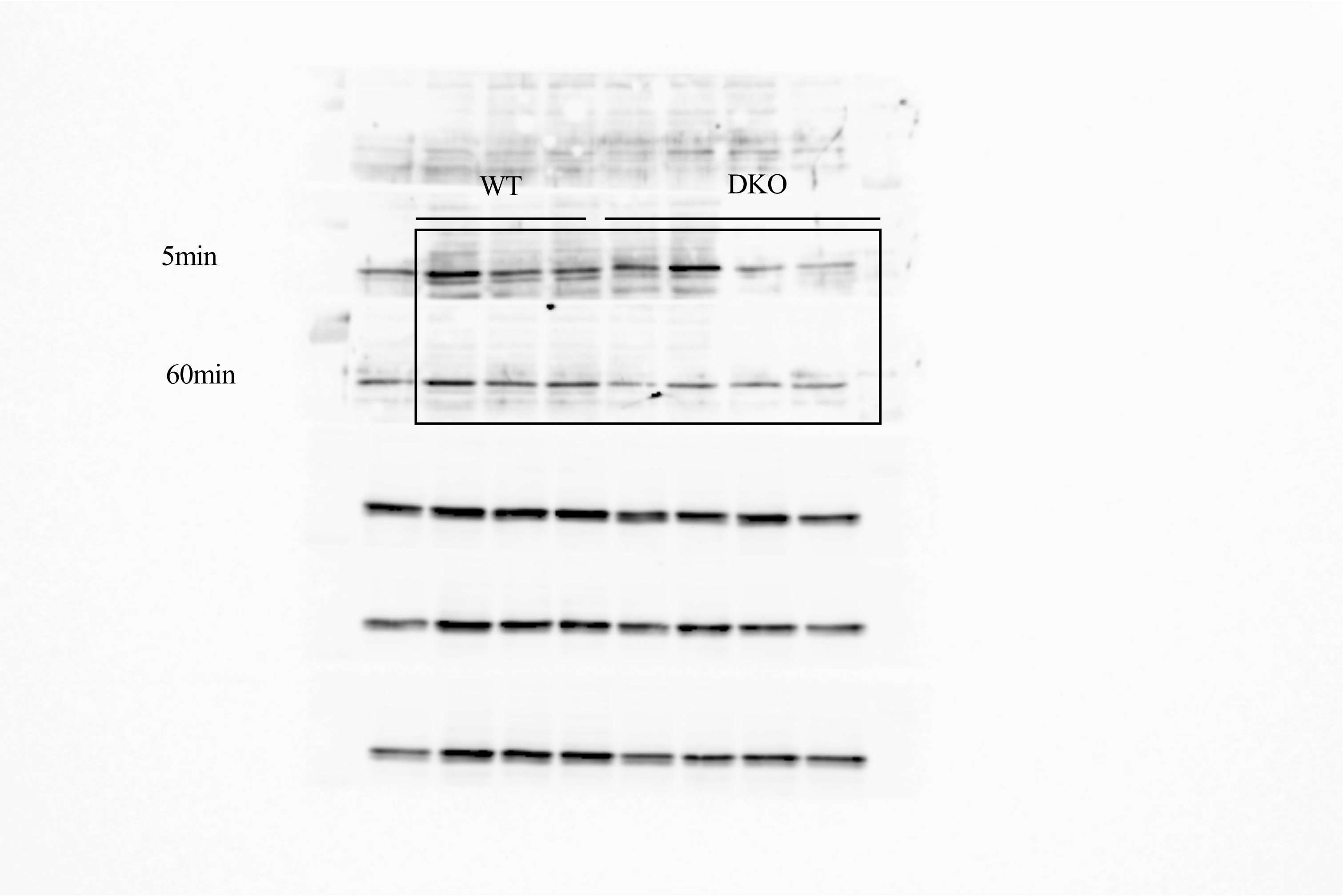

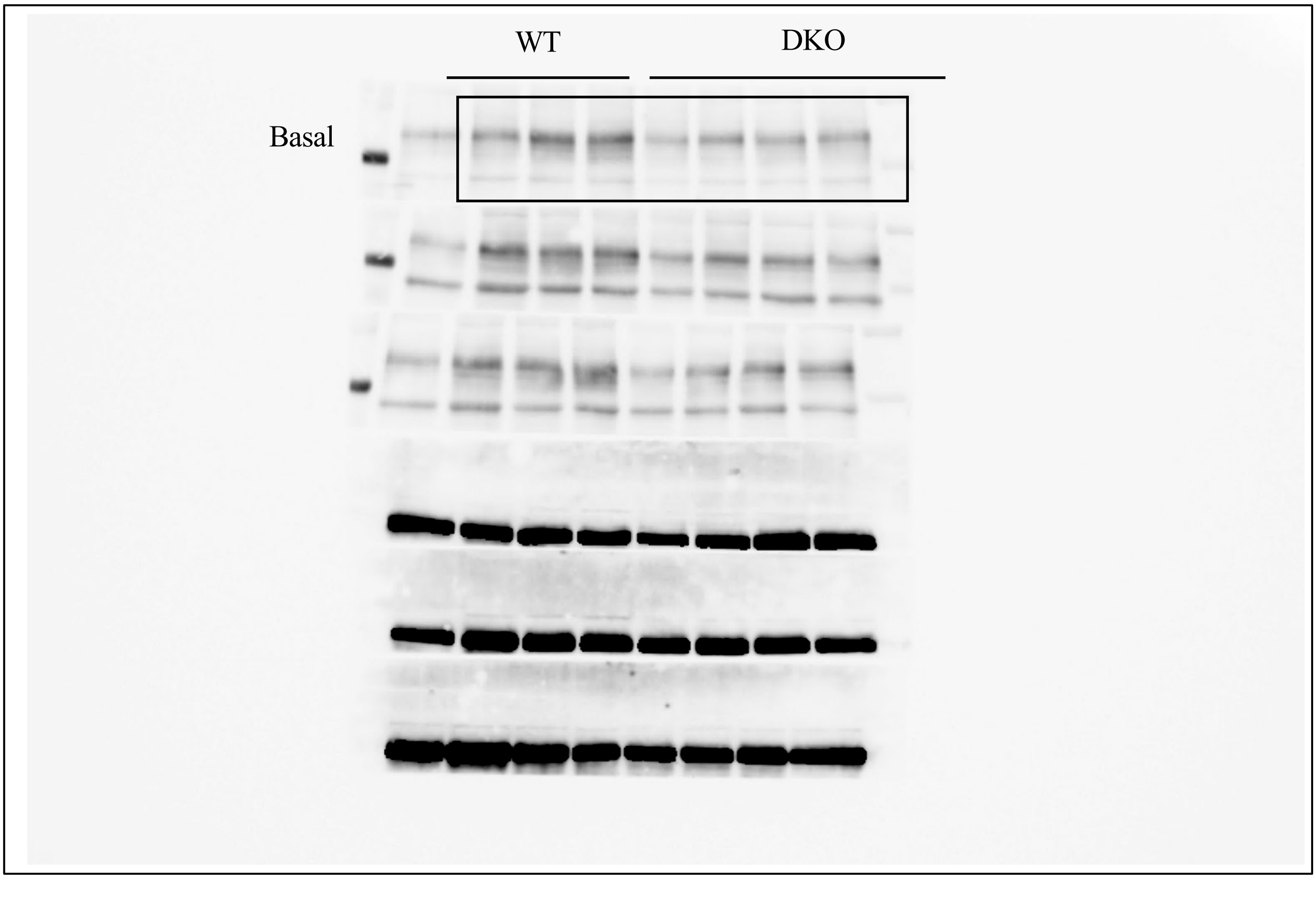

Figure 6A:
Akt

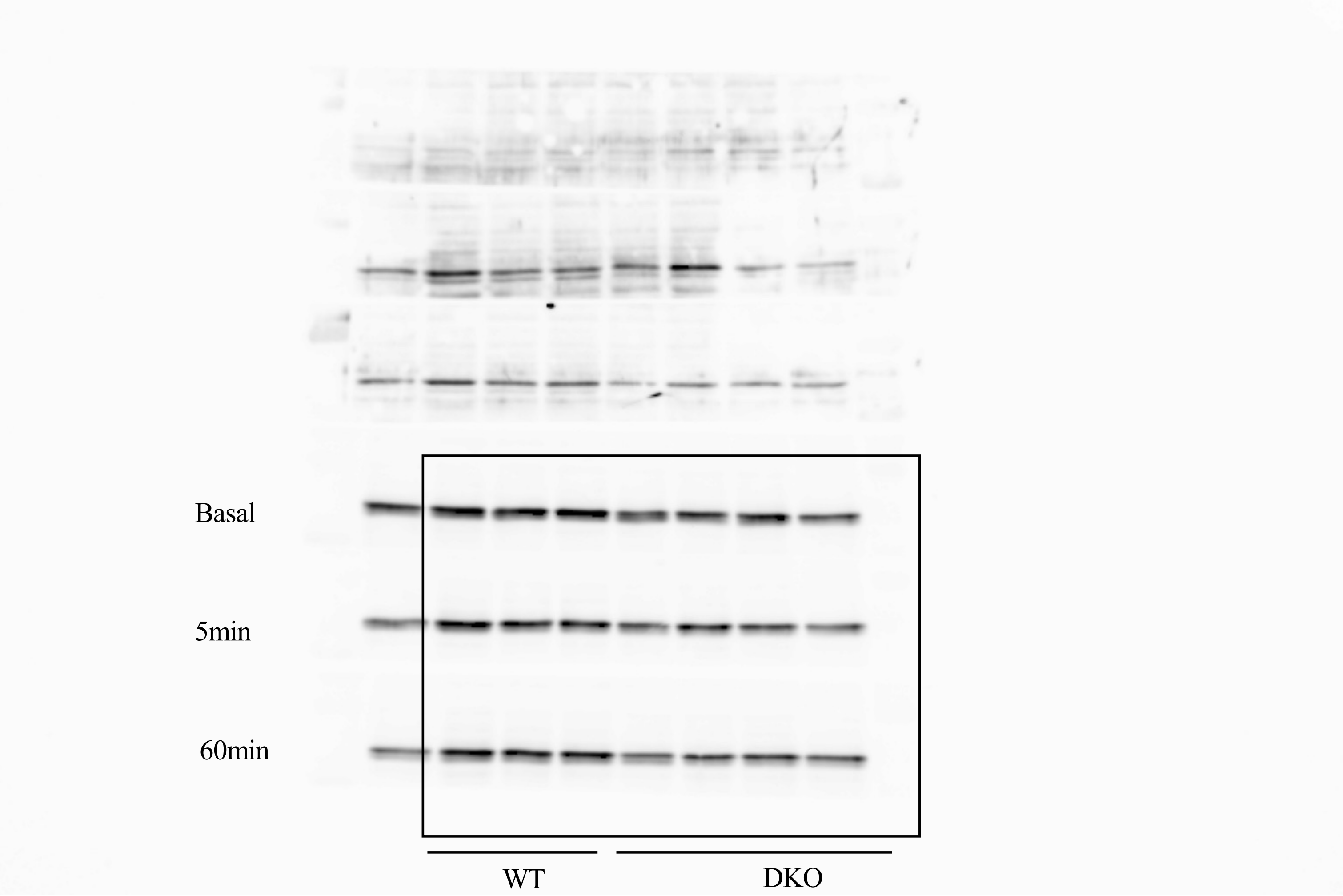

Figure 6A:
pmTOR

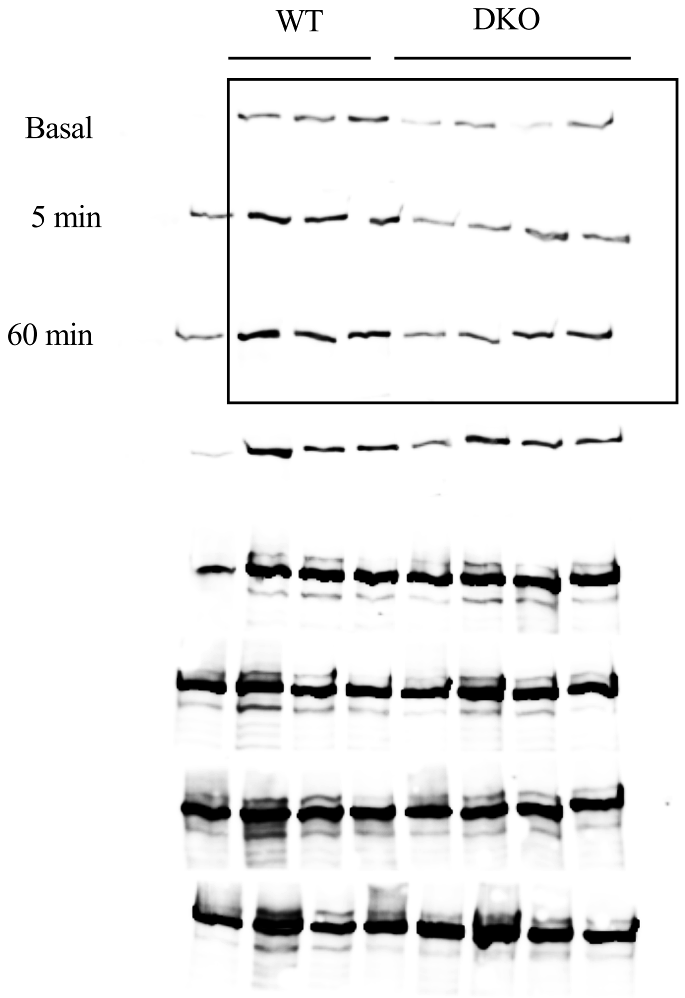

Figure 6A:
mTOR

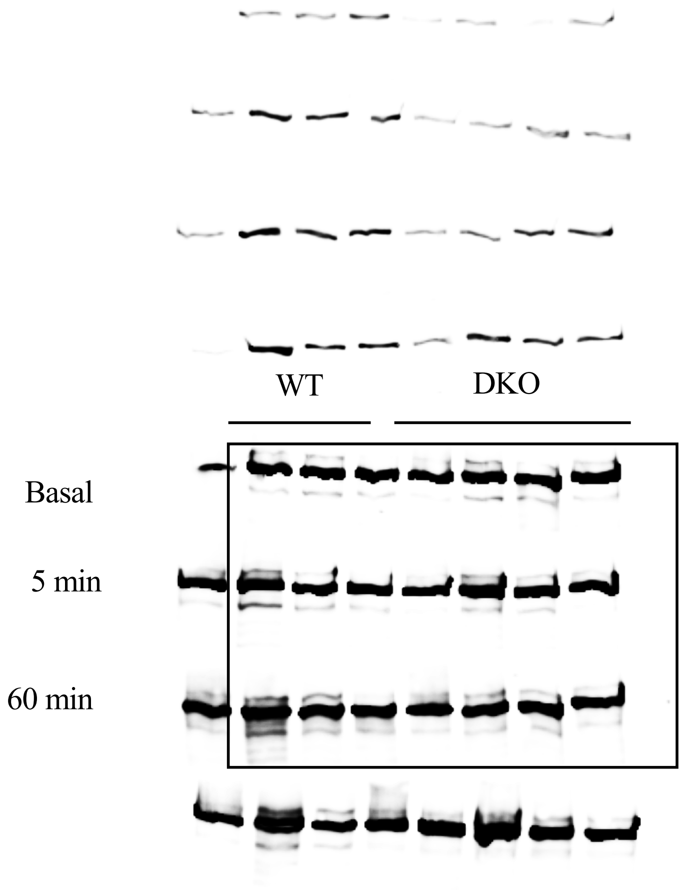

Figure 6A:
N-SREBP-1c

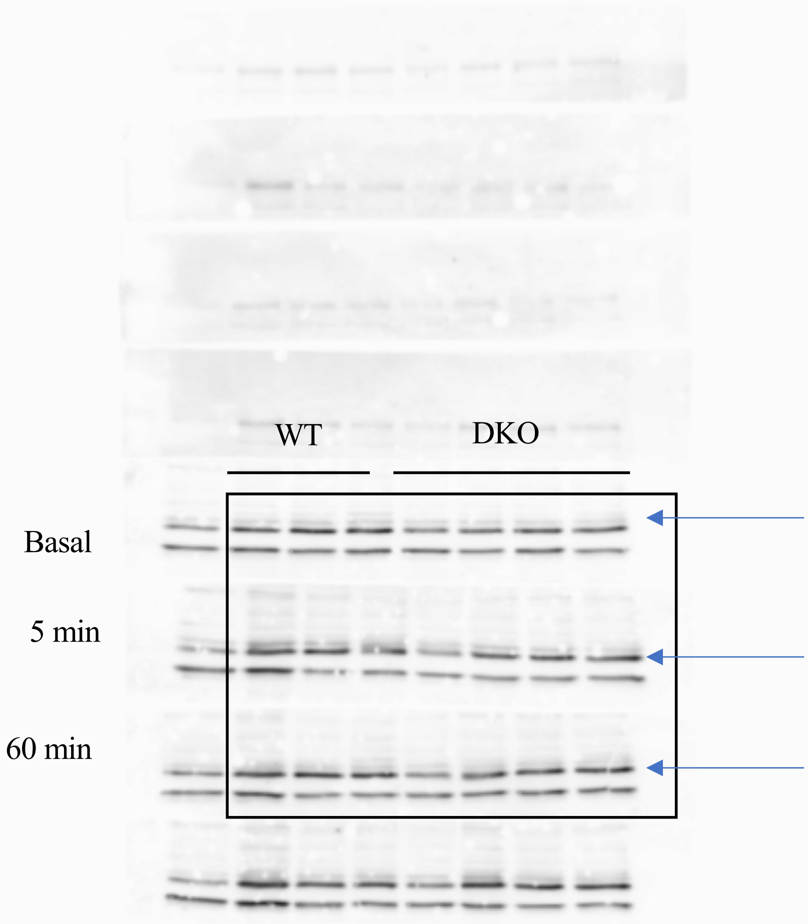

Figure 6A:
T-SREBP-1c

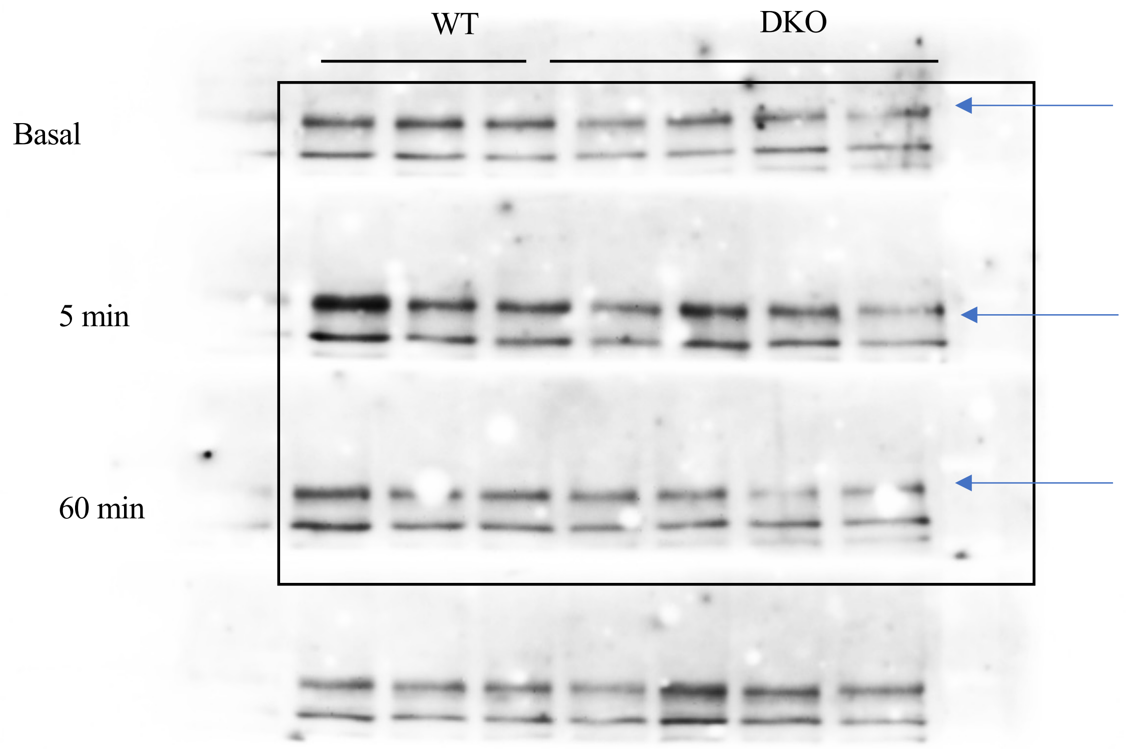

Figure 6A:
pAMPK

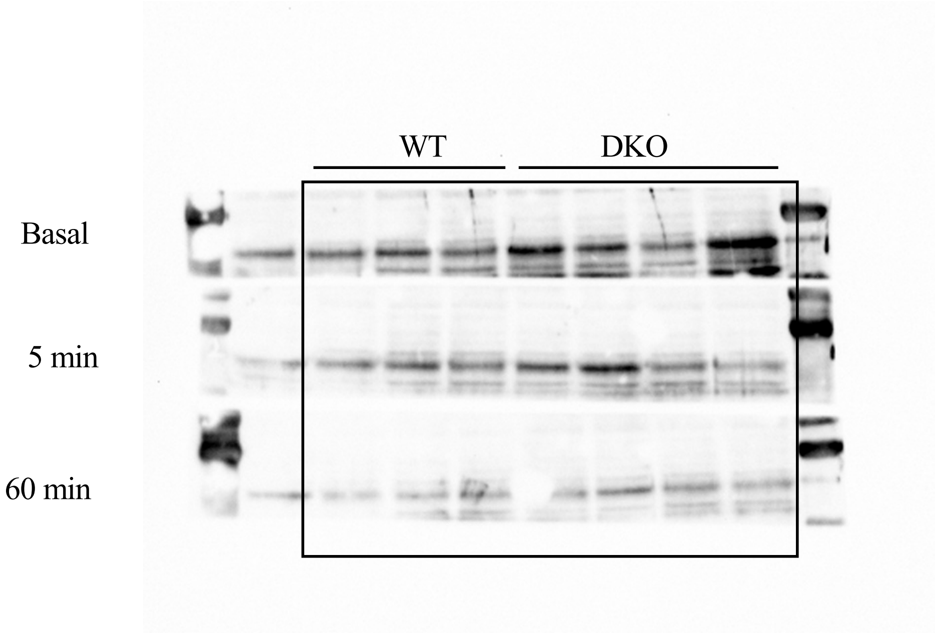

Figure 6A:
AMPK

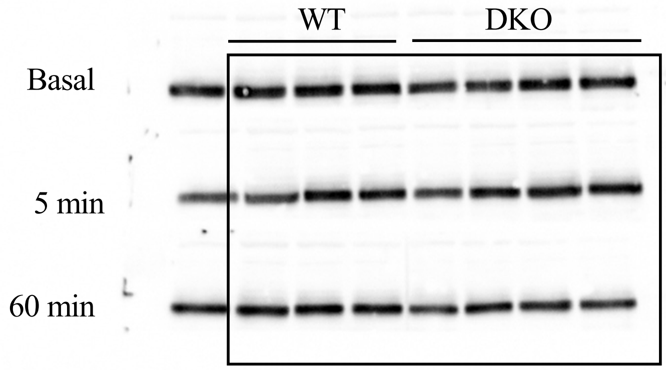

Figure 6A:
β-Actin

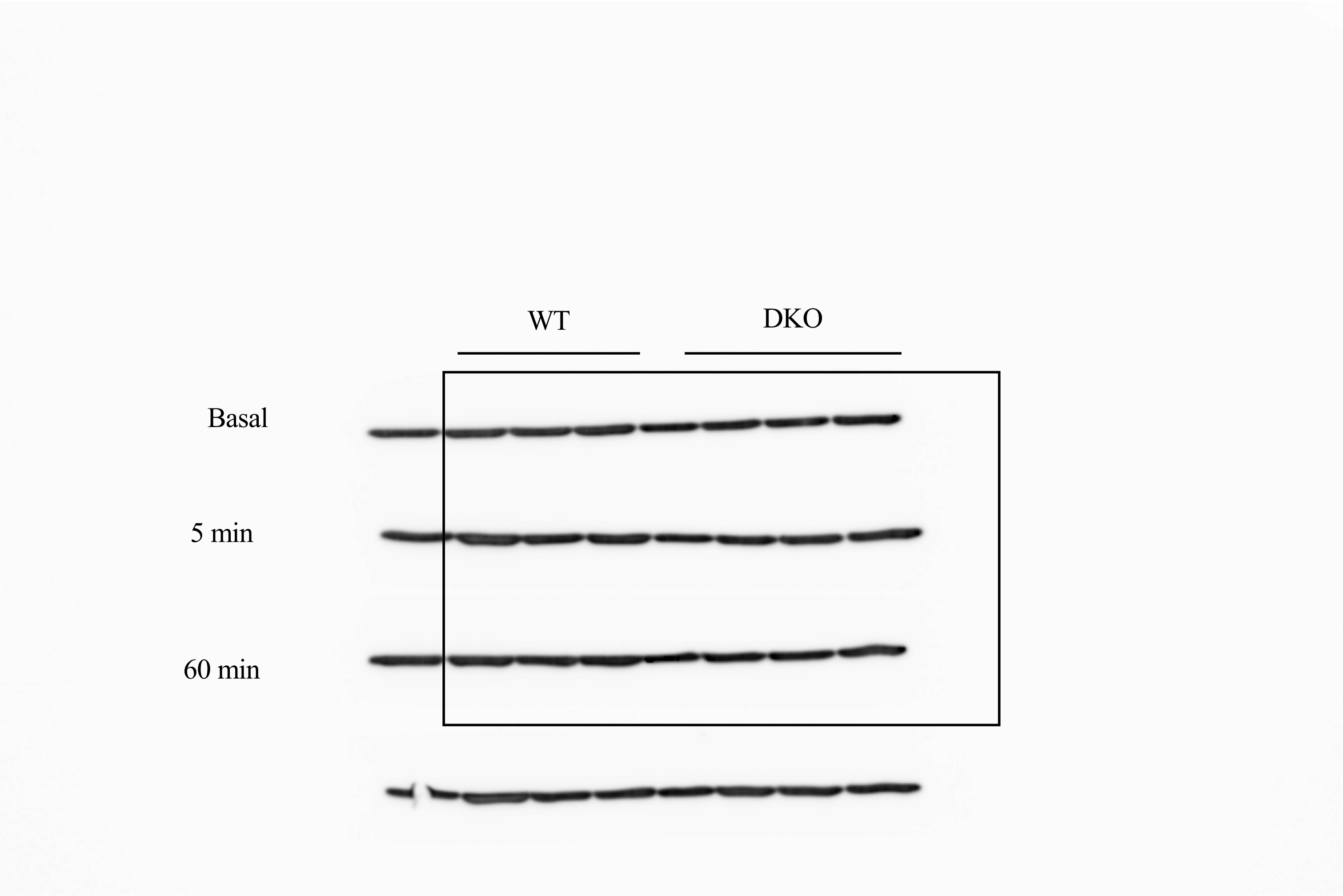

Figure 7A:
FASN

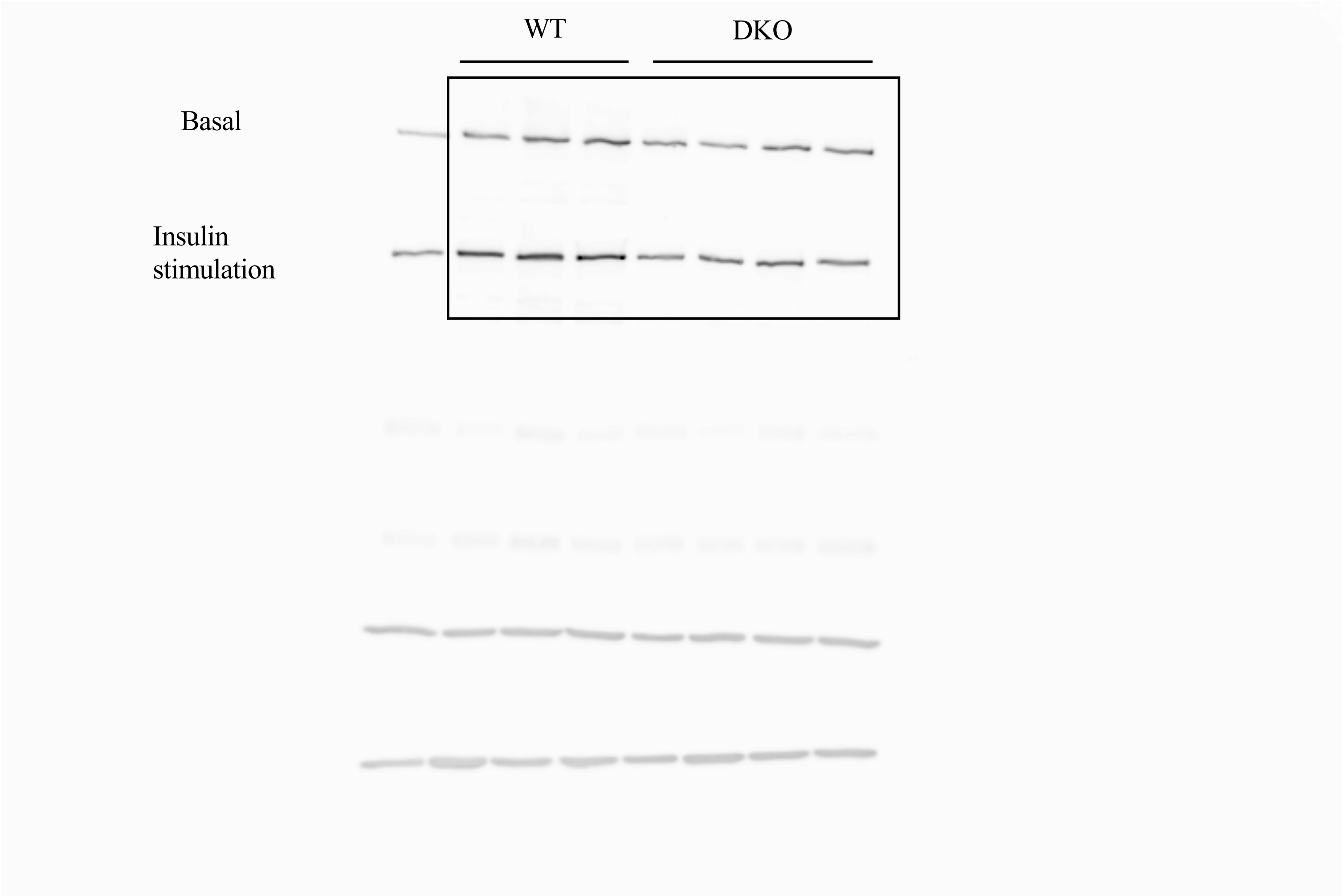

Figure 7A:
ACC

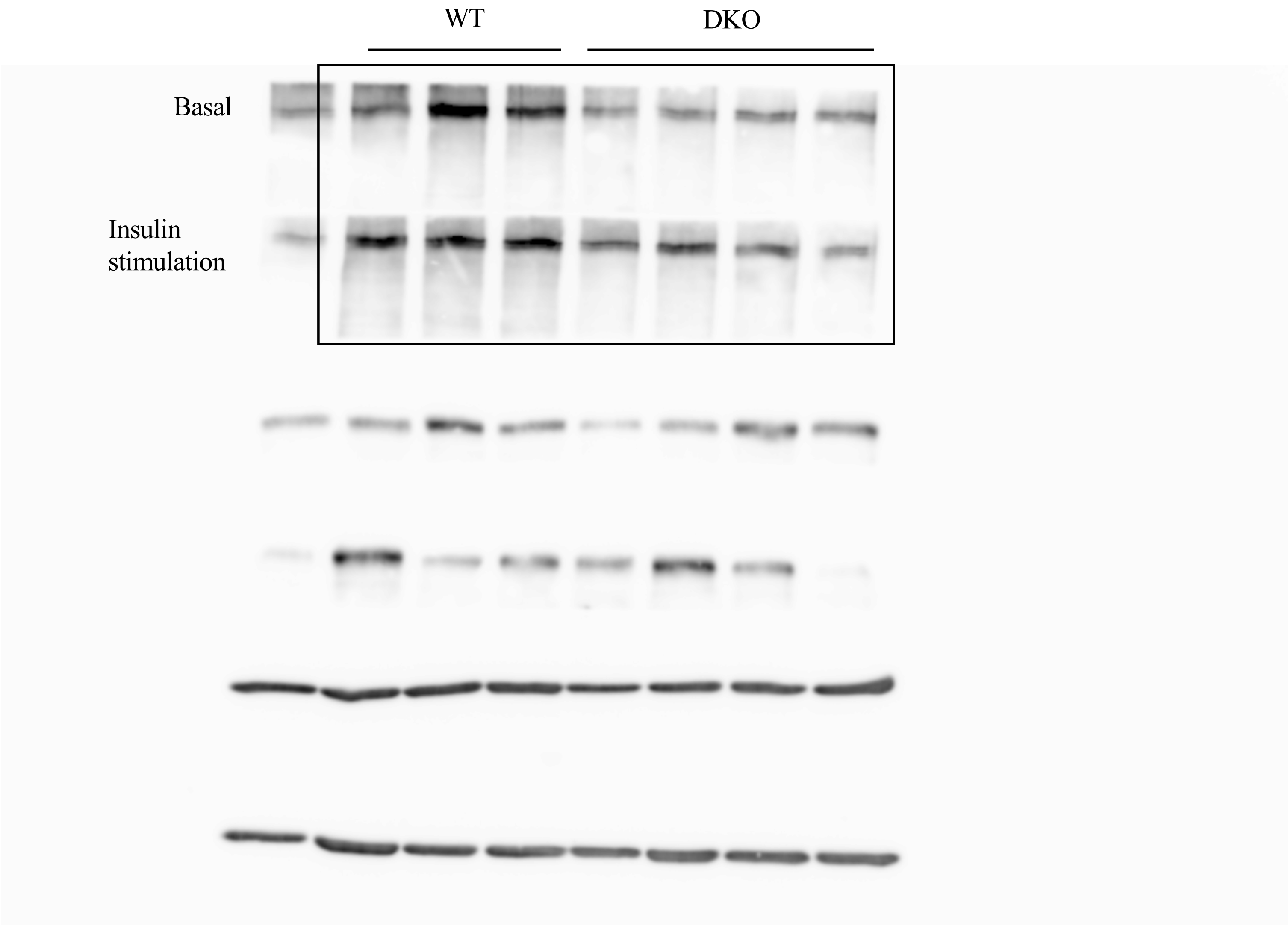

Figure 7A:
FAT/CD36

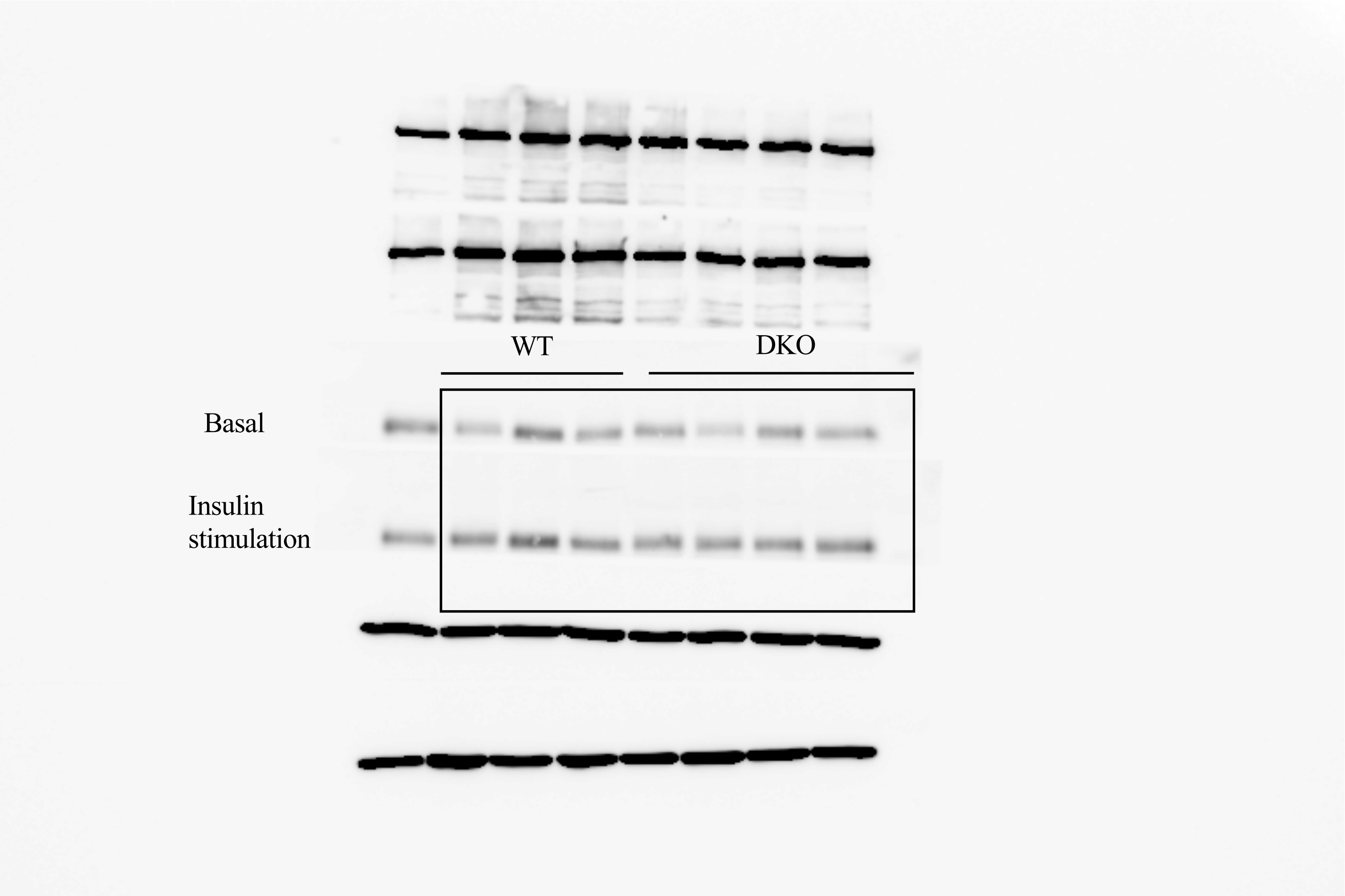

Figure 7A:
β-Actin

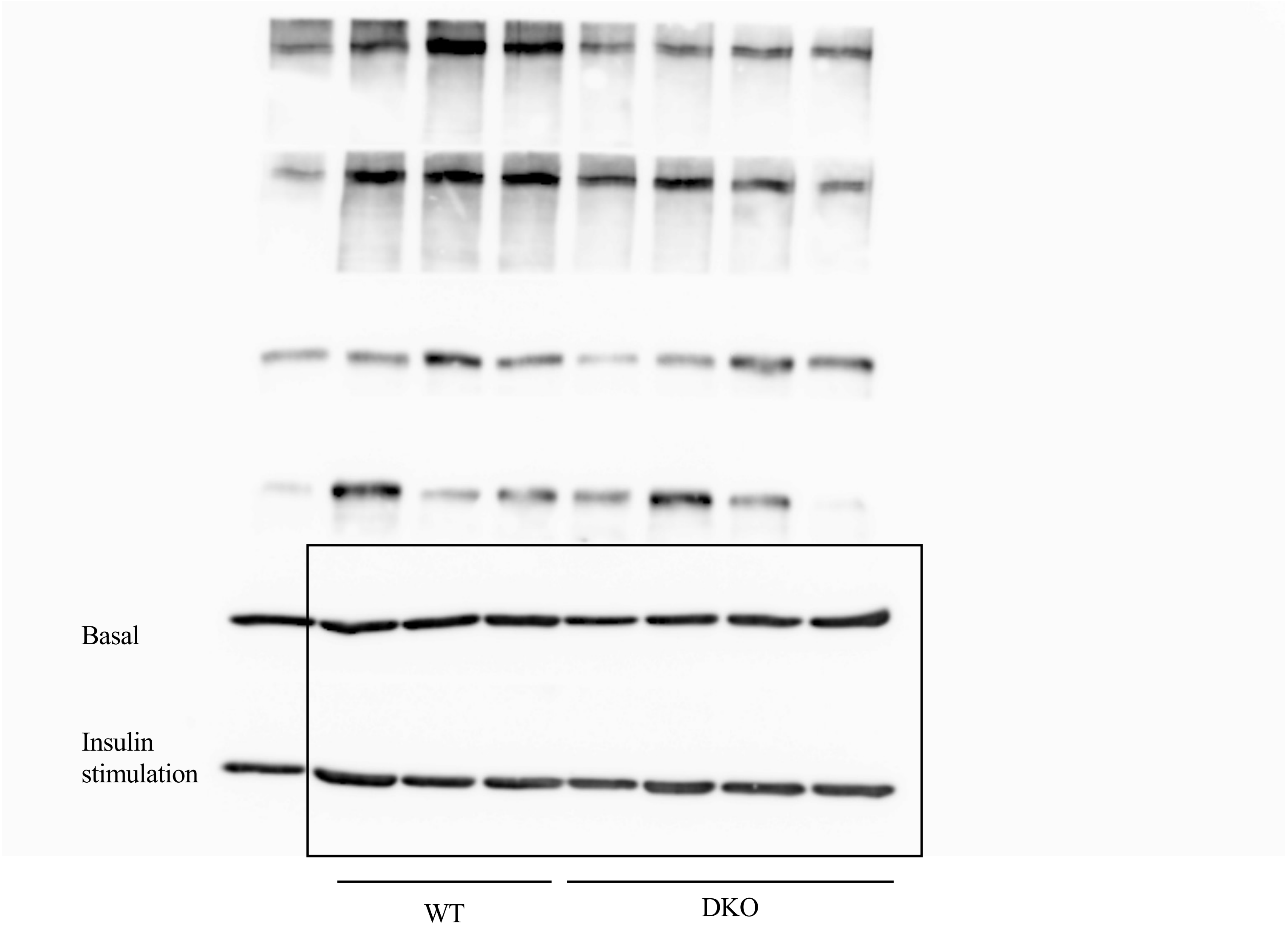

S4 Fig:
p-Tyr

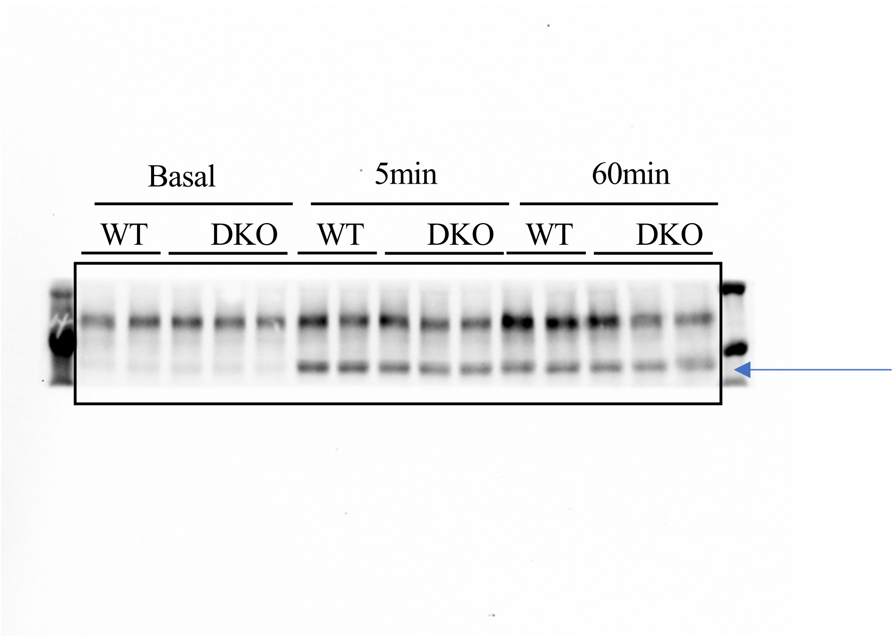

S4 Fig:
IR

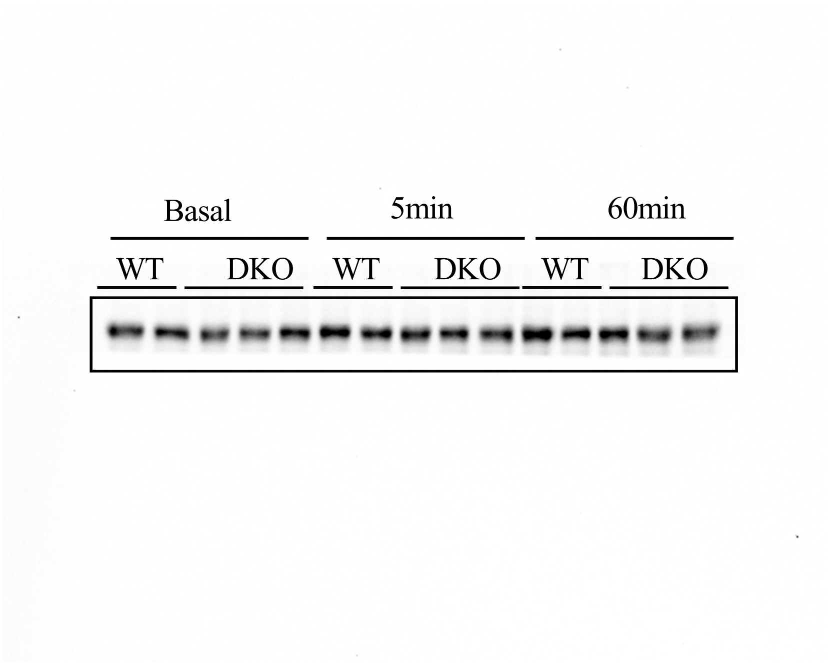

S4 Fig:
pAkt

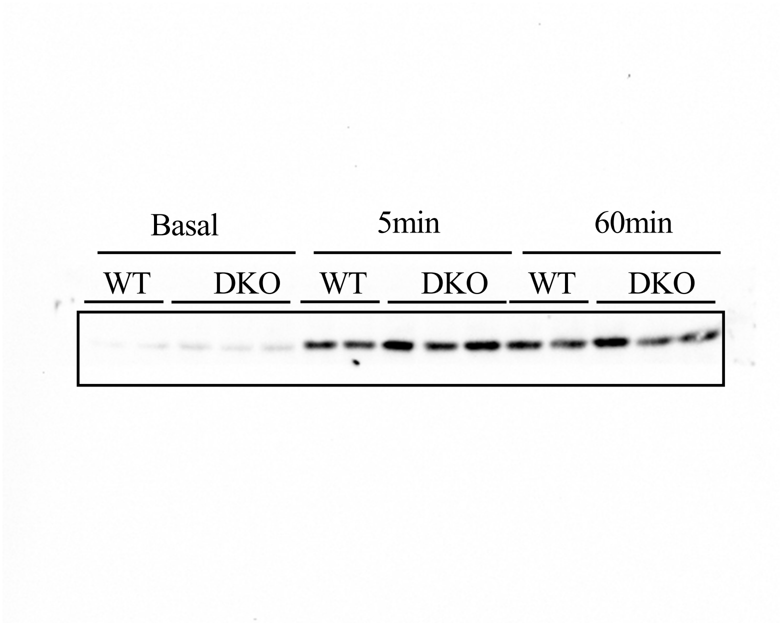

S4 Fig:
Akt

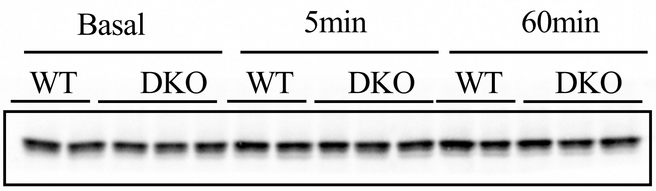

S4 Fig:
pmTOR

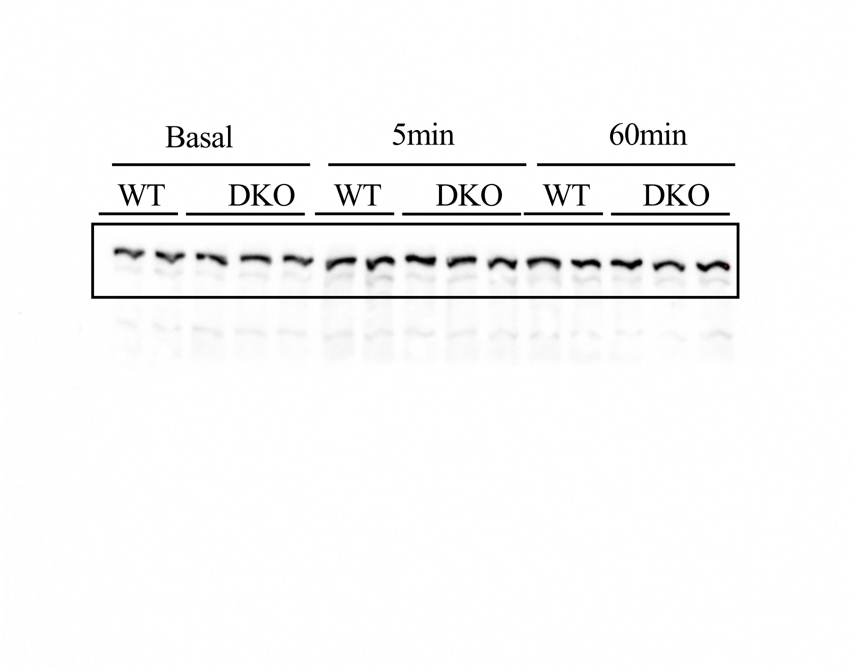

S4 Fig:
mTOR

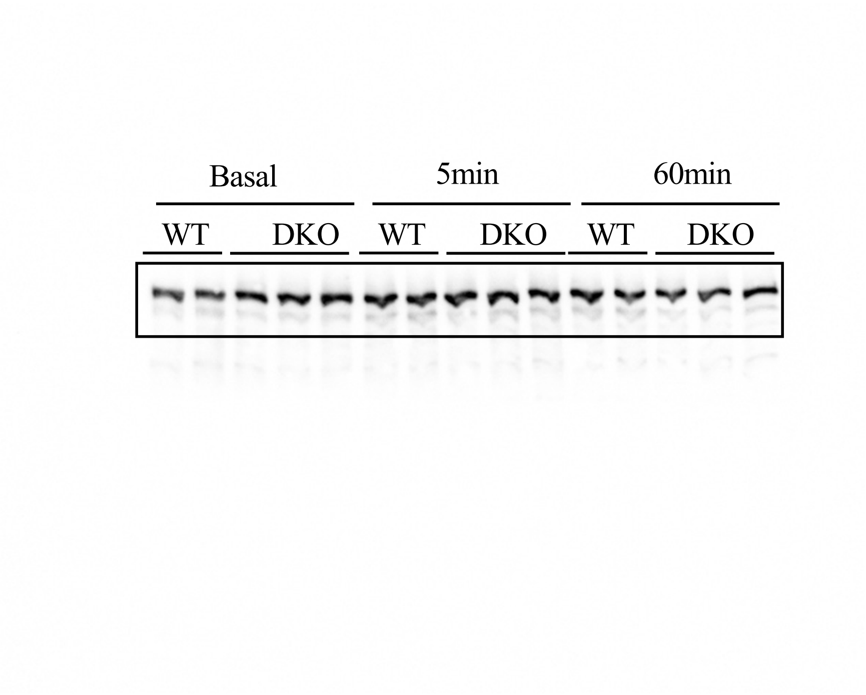

S4 Fig:
pAMPK

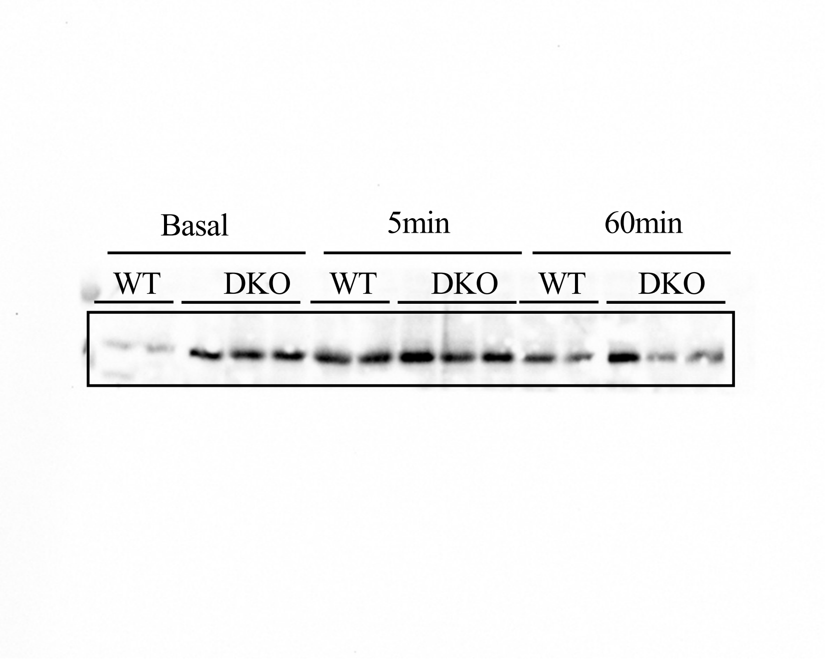

S4 Fig:
AMPK

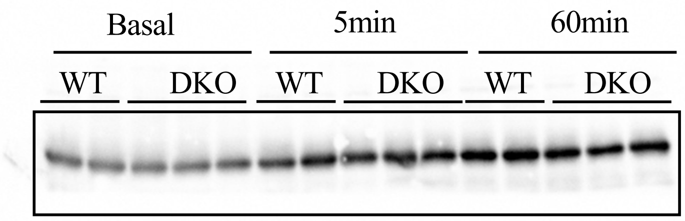

S4 Fig:
β-Actin

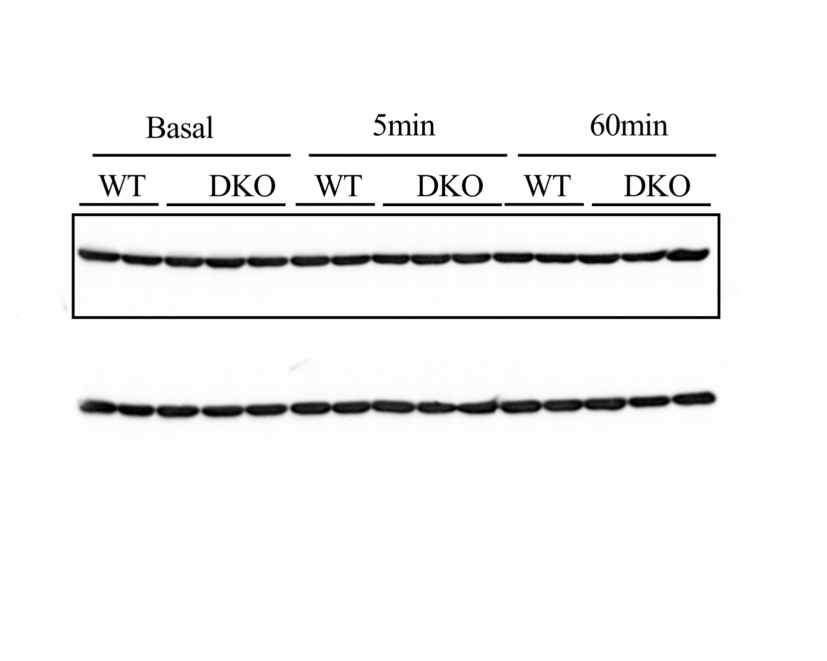

Supplement: S1 Raw images — (PDF) [file pone.0281414.s006.pdf]
